# α-Functionally Substituted α,β-Unsaturated Aldehydes as Fine Chemicals Reagents: Synthesis and Application

**DOI:** 10.3390/molecules26144297

**Published:** 2021-07-15

**Authors:** Ekaterina A. Verochkina, Nadezhda Victorovna Vchislo, Igor B. Rozentsveig

**Affiliations:** A. E. Favorsky Institute of Chemistry, Siberian Branch of the Russian Academy of Sciences, 664033 Irkutsk, Russia; vchislo@bk.ru (N.V.V.); i_roz@irioch.irk.ru (I.B.R.)

**Keywords:** α-functionally substituted α,β-unsaturated aldehydes, synthesis, organocatalysis, aldol reactions, building blocks, precursors, 2-functionally substituted 2-alkenals

## Abstract

α-Functionalized α,β-unsaturated aldehydes is an important class of compounds, which are widely used in fine organic synthesis, biology, medicine and pharmacology, chemical industry, and agriculture. Some of the 2-substituted 2-alkenals are found to be the key metabolites in plant and animal cells. Therefore, the development of efficient methods for their synthesis attracts the attention of organic chemists. This review focusses on the recent advances in the synthesis of 2-functionally substituted 2-alkenals. The approaches to the preparation of α-alkyl α,β-unsaturated aldehydes are not included in this review.

## 1. Introduction

α,β-Unsaturated aldehydes, containing a double bond conjugated with an aldehyde group, represent attractive synthetic “building blocks”. Due to the high reactivity of these polyfunctional substrates, they are widely employed, e.g., in the targeted synthesis of practically important natural compounds and other molecules (scaffolds) [1,2,3]. The presence of a functional group at the position 2 of the conjugated aldehyde system enriches chemistry of such derivatives and determines fields of their application. Some 2-functionally substituted alkenals are plant and animal metabolites [4,5,6,7,8]. In industry, α-substituted α,β-unsaturated aldehydes are used as the starting materials in the production of dyes, pesticides, aromatic compounds, and drugs [9].

Therefore, the preparation of these highly reactive substrates represents an urgent challenge for organic chemistry. It should be noted that the employment of various catalysts has expanded opportunities of 2-alkenals synthesis and sparked the interest in this area of research.

Meanwhile, many years have passed since the publication of the last review devoted to the chemistry of α-functionally substituted acrolein derivatives. Indeed, this review only covered the synthetic approaches to acrolein and its α-substituted analogs developed before 1993 [10]. There were other survey publications on this topic, in which, however, 2-functionally substituted 2-alkenals were only described partially. It is worth noting the chapter in a book [11], which only deals with the synthesis of these compounds. A review [12] was dedicated to captodative aminoalkenes, but it gave little attention to the chemistry of α-functionalized alkenals. In addition, a significant number of new original papers were published over the last decade.

**Figure 1:**
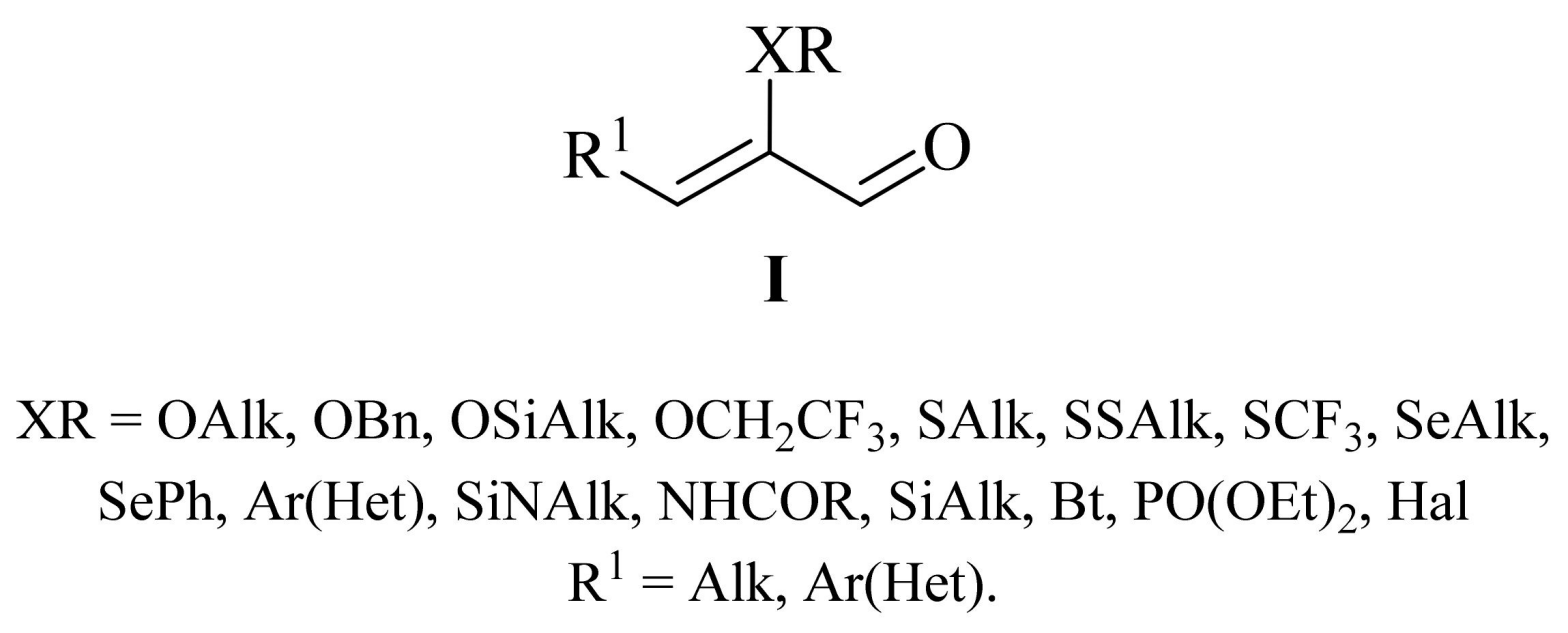
α-Functionalized alkenals.

This review summarizes methods for the synthesis of α-functionalized α,β-unsaturated aldehydes (Figure 1) bearing functional groups or halogen atoms in the position 2. Structural features, mechanistic aspects of transformations, and the prospects of these compounds’ application are discussed.

The review covers the literature published in the past 20 years. In some cases, earlier works are also considered. As the approaches to preparation of α-alkyl α,β-unsaturated aldehydes were surveyed in 2020 [13], they are not included in the present review.

## 2. Synthesis of α-Functionally Substituted α,β-Unsaturated Aldehydes

### 2.1. α-Oxygensubstituted α,β-Unsaturated Aldehydes

α-Alkoxysubstituted acrylic systems generate a particular research interest, as they represent a key structural motif of many biologically active natural compounds [14]. In addition, some derivatives of α-alkoxysubstituted α,β-unsaturated aldehydes are known to possess DNA-inhibiting and antitumor activity [15]. α-Alkoxysubstituted α,β-unsaturated aldehydes are the chemical equivalents of methylglyoxal [16], a low molecular regulator of cell growth in animals, plants, and microbes [17,18]. Derivatives of α-alkoxycinnamic aldehydes are met in plants and are structural fragments of lignin [4].

**Scheme 1:**
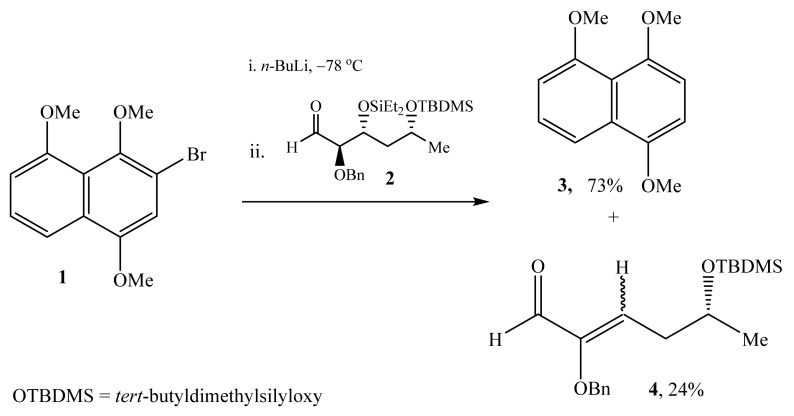
Synthesis of 5-(tert-butyldimethylsilyloxy)-2-(phenylmethoxy)-hex-2-enal (**4**). In 2001, van Boom et al. examined the transformations of D-glucose-derived eight-membered ring carbocycle **5** [20]. It was found that multistage process involved the formation of densely functional 2-benzyloxysubstituted 2-alkenals **9** (Scheme 2) via a side reaction.

The methods for the preparation of α-oxygensubstituted alkenals are limited in number. For instance, in 2000 α,β-unsaturated aldehyde **4** was obtained in 24% yield as a side product generated by beta-elimination in the reaction of trimethoxynaphthalene **1** with α-benzyloxyaldehyde **2** (Scheme 1) [19].

Aldehydes **9** were formed under the action of bases (NaOH, NaOMe) on lactone **8**, which was opened with the elimination of benzyloxy groups to furnish 2-alkenals **9** in 78–84% yields.

**Scheme 3:**
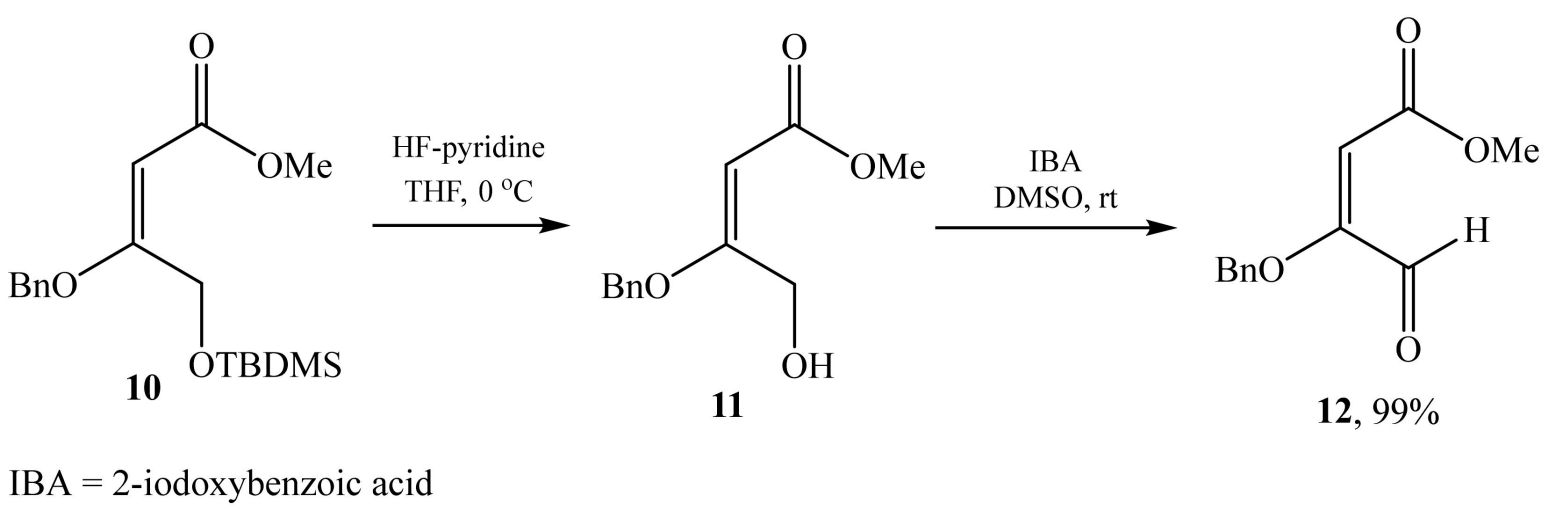
Synthesis of methyl (*E*)-3-benzyloxy-4-hydroxybut-2-enoate (**12**).

The synthesis of methyl (*E*)-3-benzyloxy-4-hydroxybut-2-enoate (**12**) from methyl (*E*)-3-benzyloxy-4-(tert-butyldimethylsilanyloxy)but-2-enoate (**10**) via an efficient two-step reaction sequence (Scheme 3) was documented [21].

The reaction involves two consecutive processes: first, the hydroxyl group is deprotected (Olah’s reagent) in THF at 0 °C or, alternatively, with HF in acetonitrile at the same temperature to give 4-hydroxybut-2-enoate (**11**). Subsequent oxidation of **11** with 2-iodoxybenzoic acid (IBA) in DMSO at room temperature affords 2-alkenal **12** in high yield (99%). It should be underlined that aldehyde **12** is an important building block in the synthesis of 3,5-unsubstituted 4-*O*-alkyl tetramates (versatile starting compounds for design of a wide variety of natural products and analogues) [22,23].

**Scheme 4:**
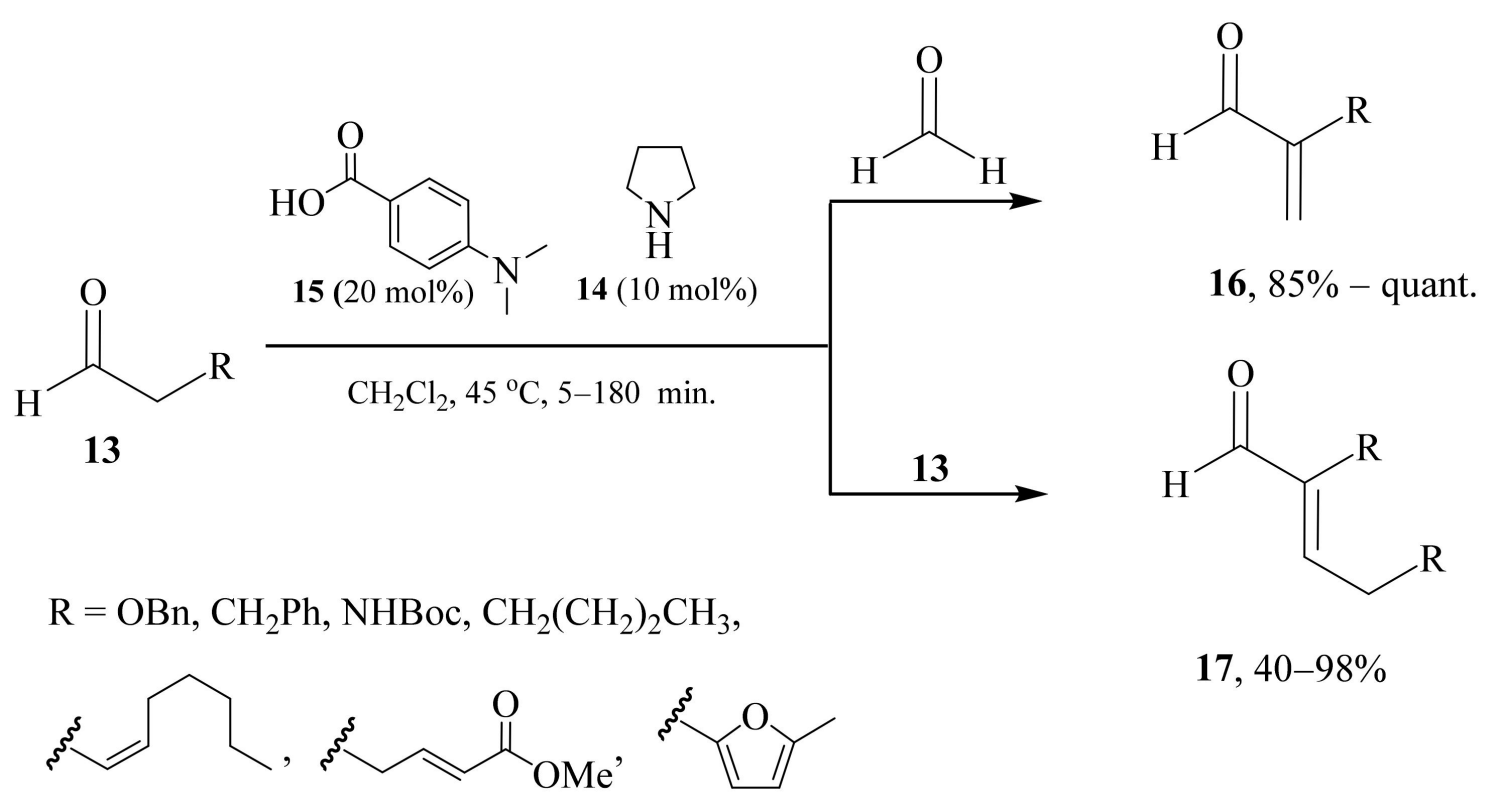
Synthesis of α-substituted α,β-unsaturated aldehydes by organocatalytic Mannich condensation of aldehydes **13**.

2-Benzyloxysubstituted 2-alkenals **16** and **17** were also obtained in good yields by an organocatalyzed aldol condensation [24,25]. Thus, the reaction of aldehyde **13** with formaldehyde in the presence of pyrrolidine **14** (10 mol%) as a catalyst and 4-*N*,*N*-dimethylaminobenzoic acid (**15**) as a cocatalyst (20 mol%) gave diverse 2-substituted 2-alkenals **16**, including 2-benzyloxysubstituted 2-alkenals, in high yield. In the absence of formaldehyde at the same conditions, the catalytic system promoted the self-condensation reaction of **13** to produce aldehyde **17**. The yields of products **17** reached 98% (Scheme 4).

**Scheme 5:**
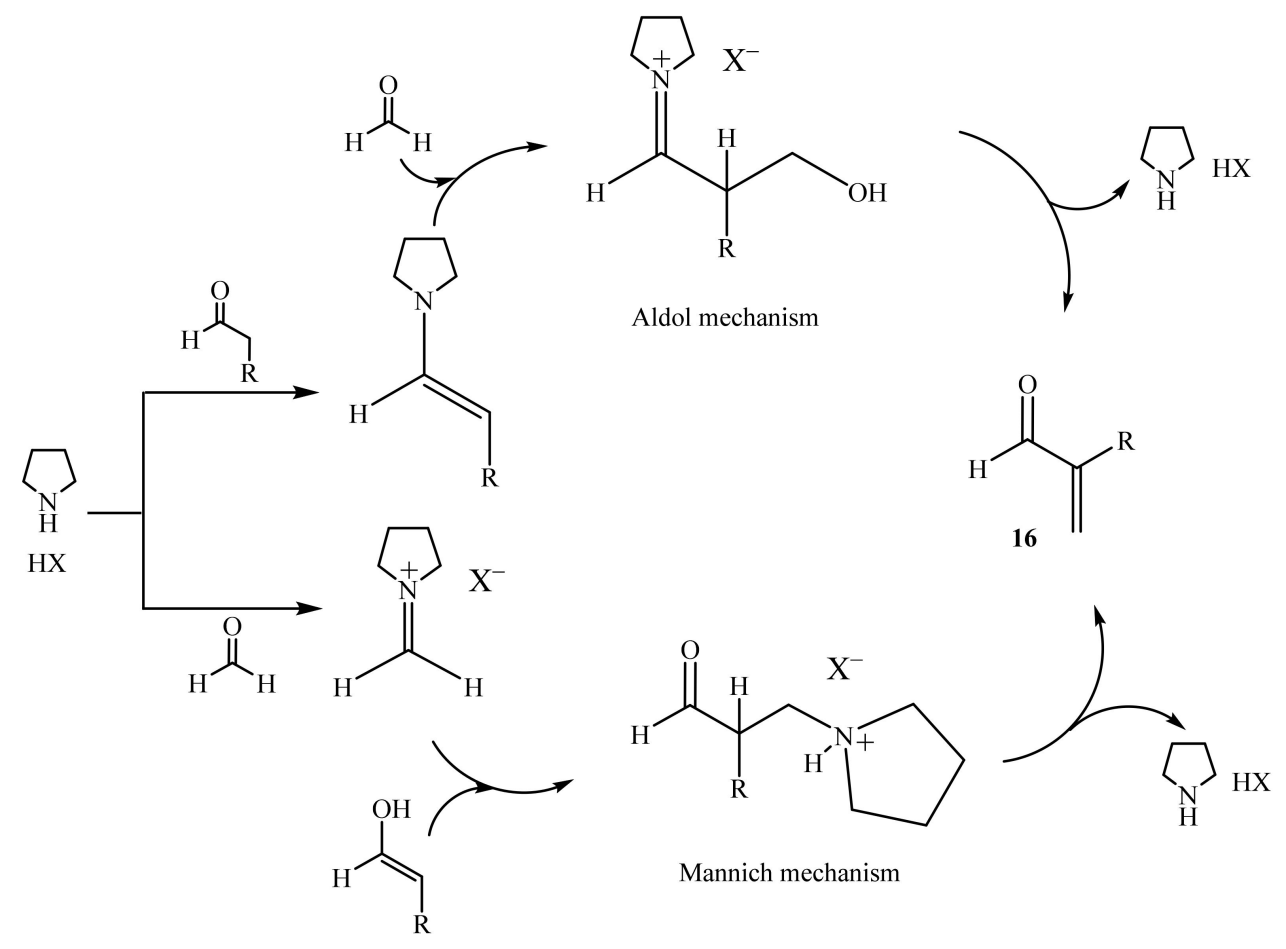
Possible mechanisms for the α-methylenation reaction.

It is assumed that the reaction course is determined by the kinetic resolution of the catalyst between formaldehyde and other aldehydes. Presumably, the formation of formaldehyde/catalyst complex (or a covalent intermediate) is more preferable than the formation of the related compounds with other aldehydes. The mechanism of the Mannich reaction suggests that the enol form of donor aldehyde reacts with an iminium compound produced by the acceptor aldehyde (usually formaldehyde) (Scheme 5).

**Scheme 6:**
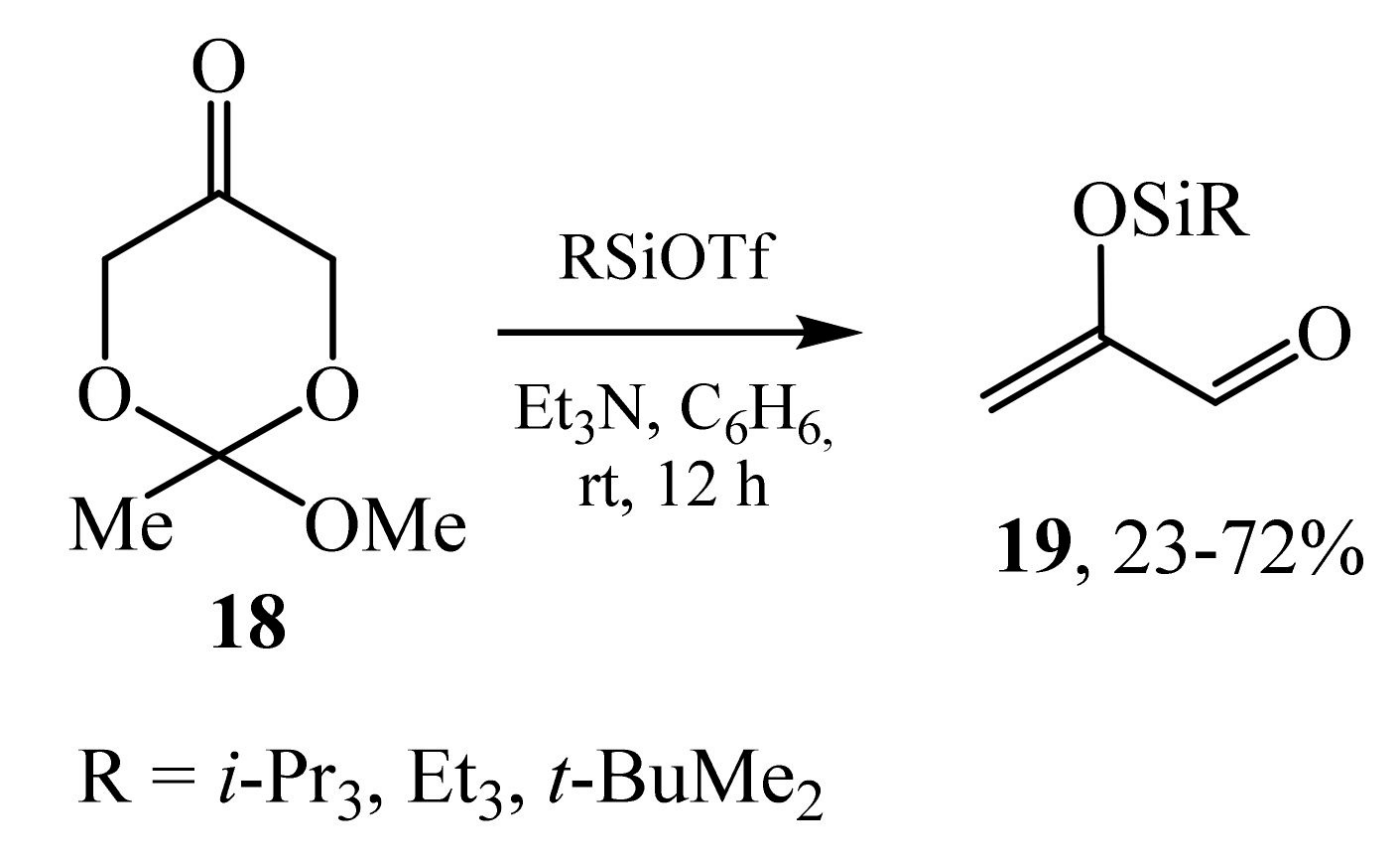
Synthesis of 2-(trialkylsilyloxy)-2-propenals **19**.

The α-siloxysubstituted α,β-unsaturated aldehydes are widely employed in organic synthesis as dienophiles for (4+3) cycloaddition reactions in the presence of Lewis acid [26]. The reactivity of such aldehydes was first tested by Sasaki et al. in the construction of various cycloadducts [27]. In 2000, Harmata and Sharma [28] obtained 2-(trialkylsilyloxy)-2-propenals **19** from trialkylsilyltriflates and 2-methoxy-2-methyl-[1,3]-dioxan-5-one **18** by the retro-hetero-Diels–Alder reaction (Scheme 6). The target alkenals were further involved in the reaction with dienes (furan, cyclopentadiene, 1,3-butadiene).

It is worthwhile to note that the trialkylsilyl group is perfectly retained in the obtained cycloadducts.

**Scheme 7:**
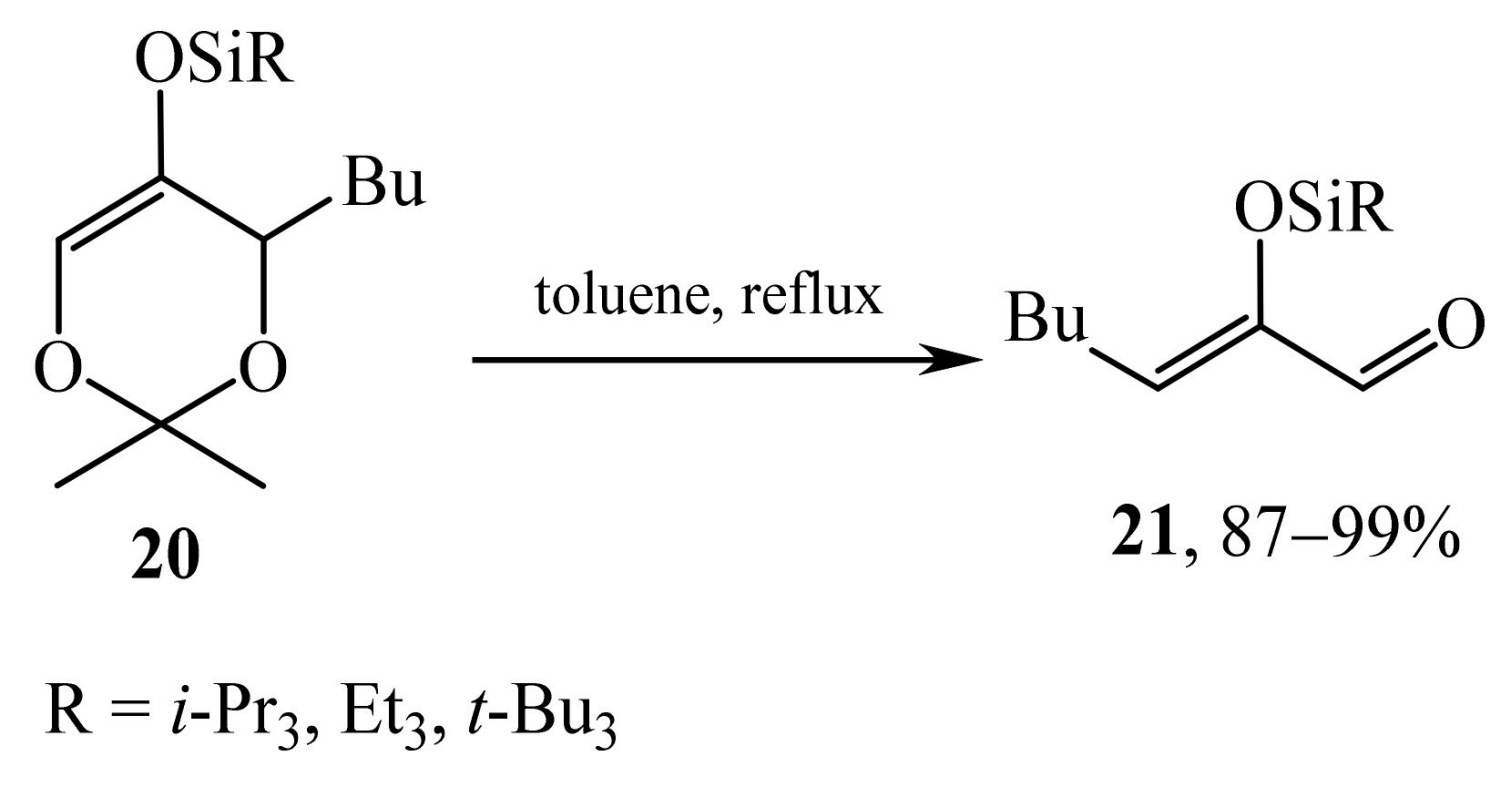
Retro-hetero-Diels–Alder synthesis of α-siloxysubstituted α,β-unsaturated aldehydes **21**.

Similar to the above-described reactions, 2-(trialkylsilyloxy)-2-enals **21** were synthesized from 4-butyl-5-(trialkylsilyloxy)-2,2-dimethyl-4*H*-[1,3]-dioxins **20** upon refluxing in toluene [29] (Scheme 7).

The yields of α-(trialkylsilyloxy)substituted α,β-unsaturated aldehydes **19**, **21** reach 72–99%. However, these compounds are extremely unstable in moist air. α-(Trialkylsilyloxy)acroleins **21**, unlike compounds **19**, contain a substituent in the β-position that promotes a more efficient Sasaki-type [4+3] cyclization with dienes in the presence of Lewis acids with excellent regio- and/or stereoselectivity. Enal, with a similar structure to **21**, has been used for the synthesis of (±)-Cortistatin *J* (novel steroidal alkaloid) [30].

**Scheme 8:**
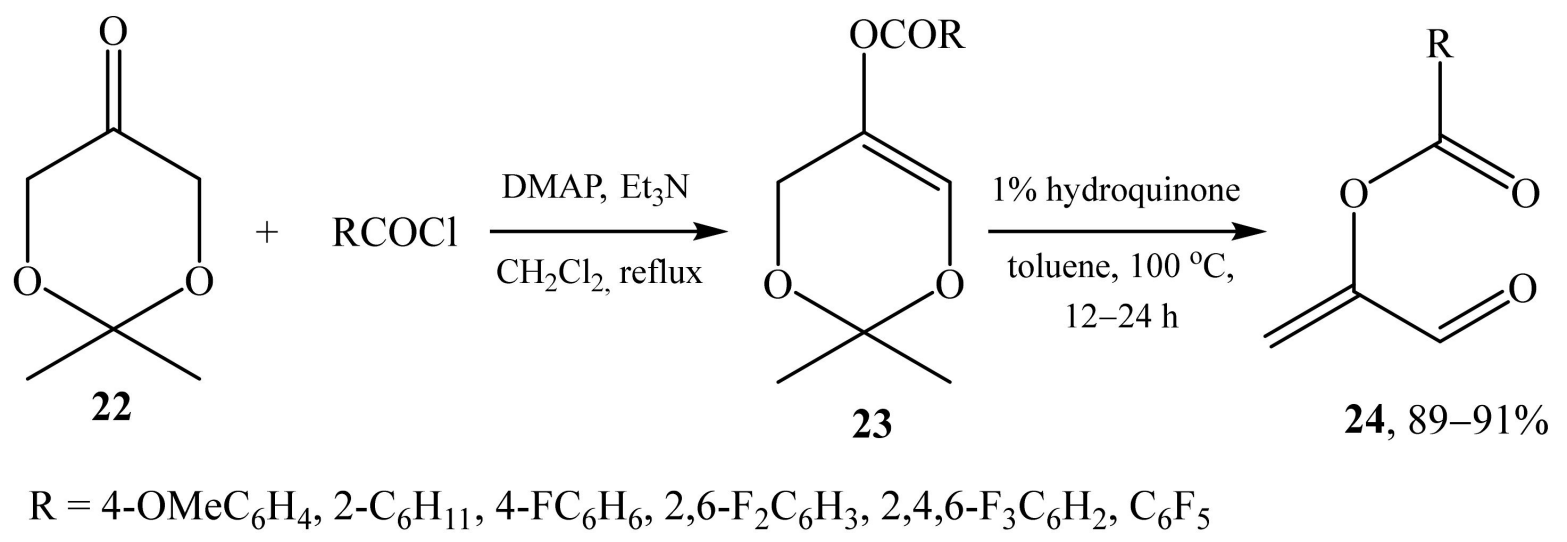
Synthesis of 2-acyloxysubstituted 2-propenals **24**.

The retrocycloaddition reactions of 1,3-dioxins **23** [31] proved to be efficient for the synthesis of 2-acyloxyacroleines **24** in the presence of 1% hydroquinone at 100 °C in toluene under an argon atmosphere (Scheme 8). These aldehydes **24** can be purified using column chromatography. However, substance polymerize easily upon standing at room temperature and are best stored at low temperatures (−20 °C).

2-Acyloxysubstituted 2-propenals **24** are widely employed in [2+2]-cycloadditions [32] and Diels–Alder reactions [33,34,35,36,37,38].

**Scheme 9:**
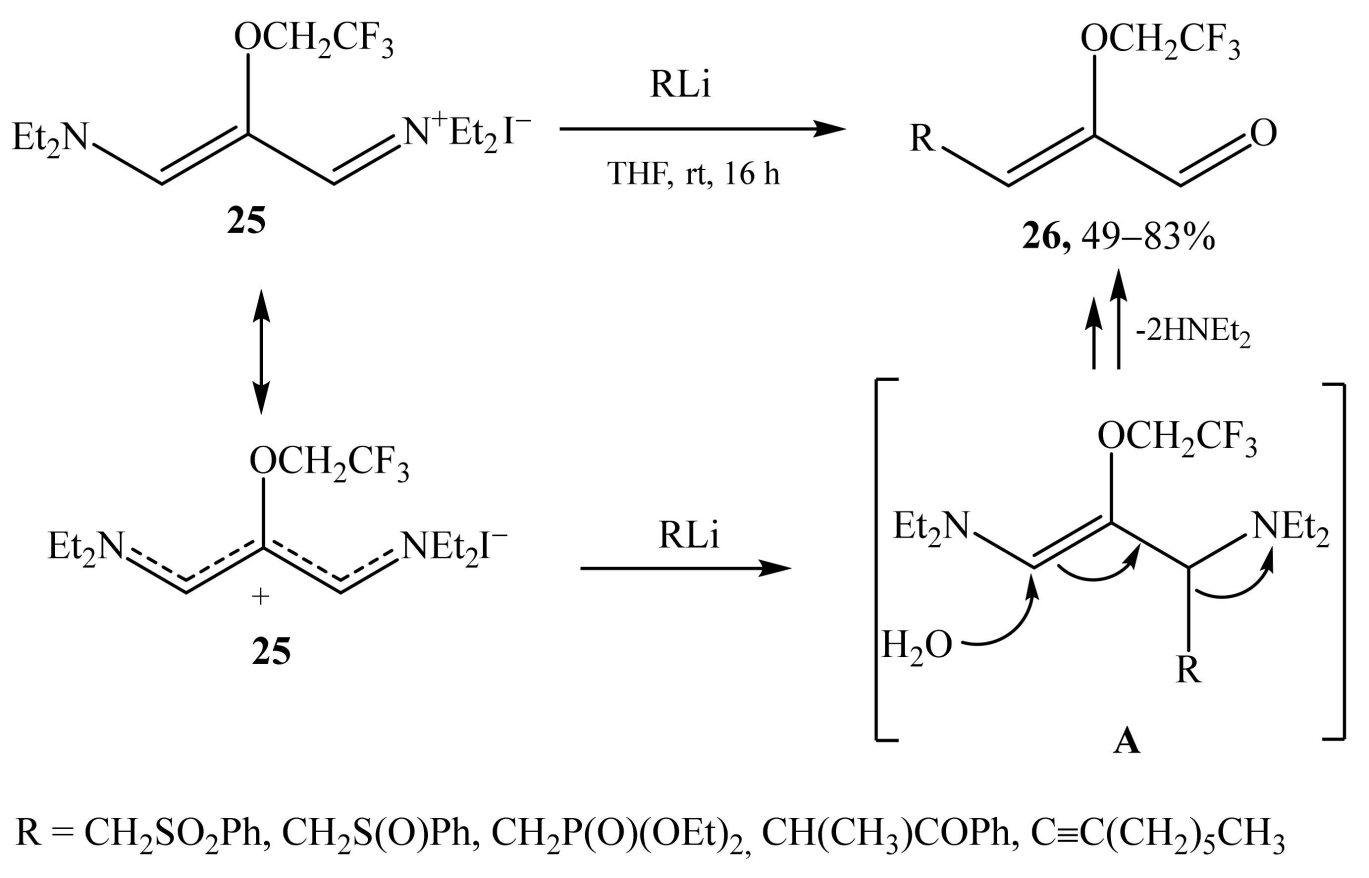
Synthesis of α-trifluoroethoxysubstituted α,β-unsaturated aldehydes **26**.

The compounds containing the fluoroalkoxy group are used as pharmaceuticals, for example, Flecainide and Lansoprazole [39]. 2-Trifluoroethoxysubstituted alkenals **26** were obtained by Yamanaka et al. [40] through the reaction of β-trifluoroethoxyvinamidinium salts (**25**) with lithium acetylide and other carbanions derived from methyl compounds bearing sulfonyl, sulfinyl, and phosphonyl groups (Scheme 9).

Mechanistically, the reaction is triggered by the attack of carbanion at the carbon atom of vinamidinium salt **25** to generate a tetrahedral intermediate **A**. When the hydrogen atom at the α-position to the diethylamino group in substituent R is weakly acidic, and/or sterically hindered, and the base cannot attack it, the diethylamino group is not cleaved from the intermediate **A**. Subsequent hydrolysis leads to elimination of the diethylamino group to give aldehydes **26** via the formation of N,O-acetal.

**Scheme 10:**
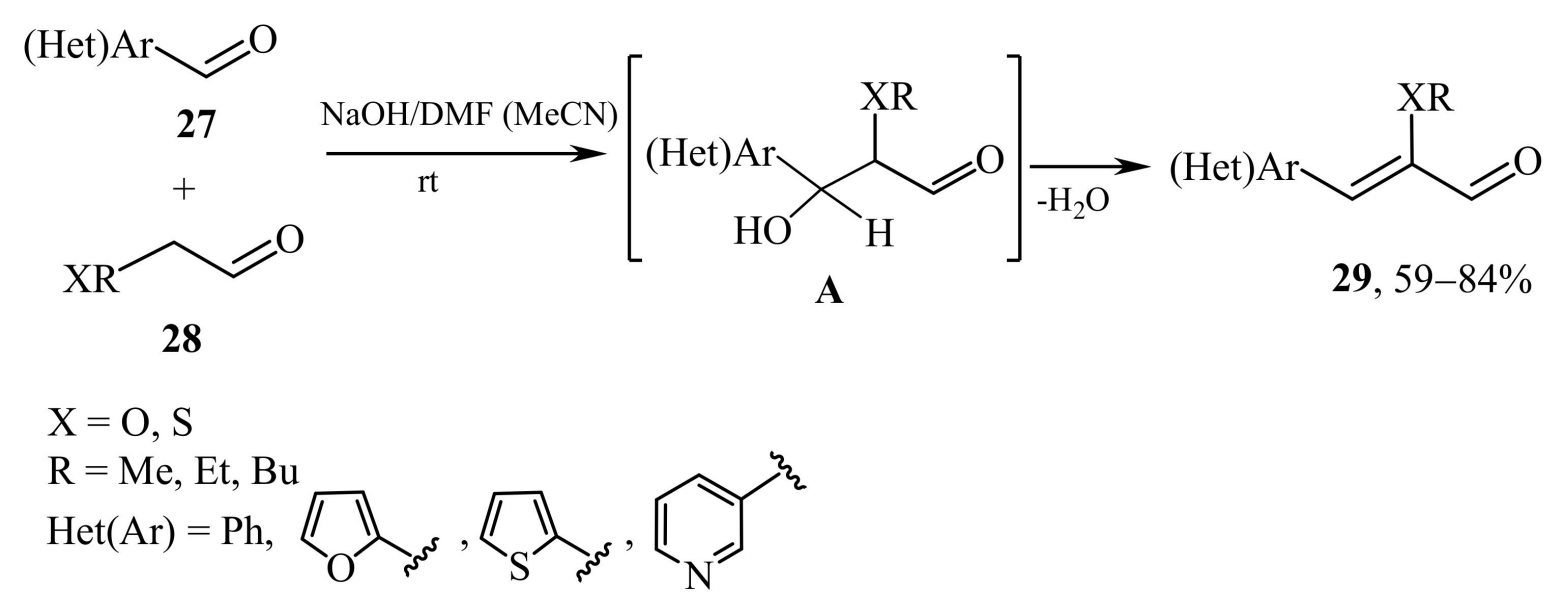
Synthesis of α-alkoxy- and α-alkylthiosubstituted α,β-unsaturated aldehydes by condensation in two-phase systems.

Heteroaromatic α-alkoxy- and α-alkylthiosubstituted α,β-unsaturated aldehydes **29** were prepared using alkaline catalysts in the two-phase systems (Scheme 10) [41].

The aldol condensation proceeds stereoselectively to deliver the *Z*-isomer. Unlike classical protocols using alkali [42,43,44], heterogenous system solid NaOH/DMF(MeCN) allows the avoidance of by-products and increase in yields of the target compounds. α-Alkoxy- and α-alkylthiosubstituted α,β-unsaturated aldehydes **29** are valuable building blocks in the synthesis of oxazoles, pyrroles, and quinoxalines [45].

**Scheme 11:**
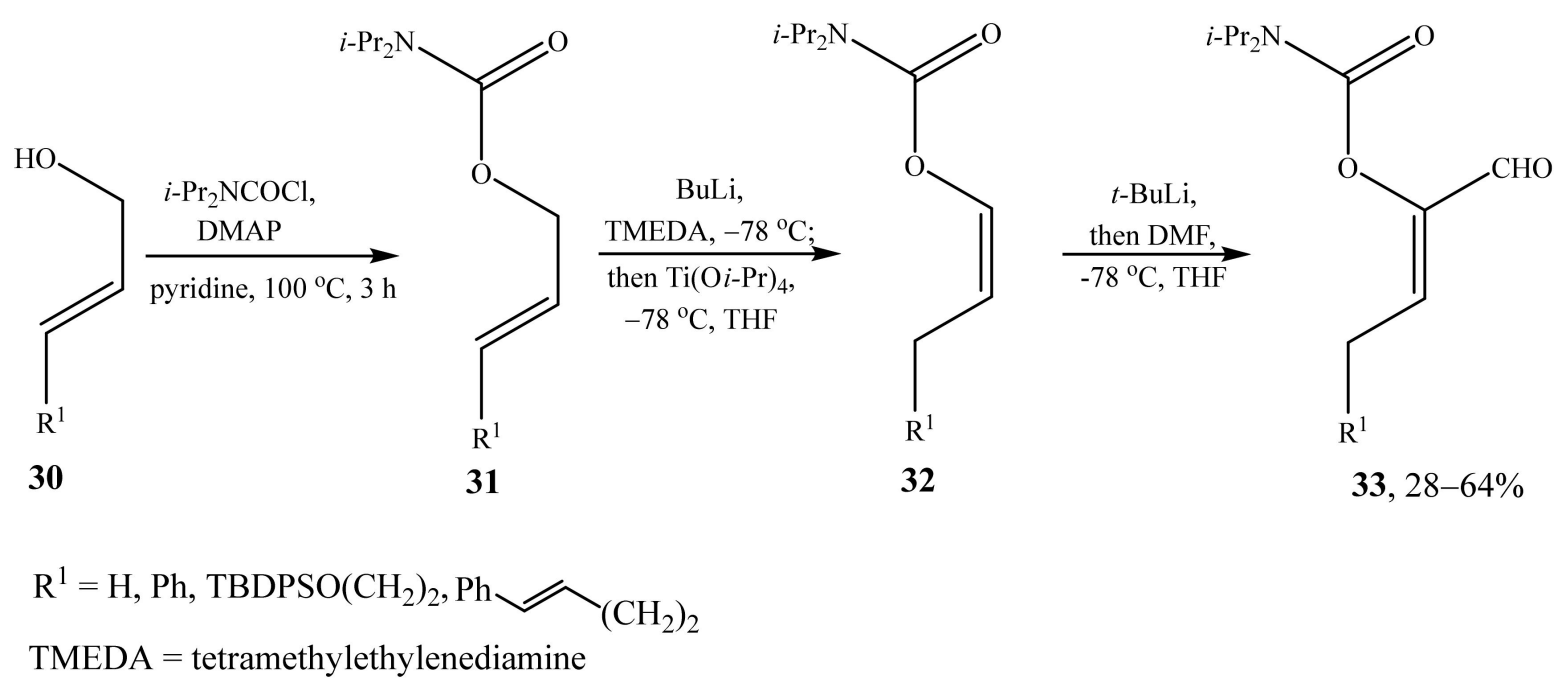
Synthesis of α-(*N*,*N*-diisopropylcarbamoyloxy)-β-alkylacroleins **33**.

In 2012, K. Ishihara synthesized α-(*N*,*N*-diisopropylcarbamoyloxy)-β-alkylacroleins **33 [46]**. The synthesis of **33** starts with carbamoylation of allylic alcohols **30** with diisopropylcarbamic chloride (*i*-Pr_2_NCOCl) in the presence of a catalytic amount of 4-dimethylaminopyridine (DMAP) (Scheme 11).

The carbon–carbon double bond migration of the allylic carbamates **31** successfully gave the corresponding *Z*-vinyl carbamates **32** as single stereoisomers. Formylation of the vinyl carbamates **32** with *t*-BuLi and *N*,*N*-dimethylformamide (DMF) afforded (*Z*)-α-(N,N-diisopropylcarbamoyloxy)-β-alkylacroleins **33** as single stereoisomers in the total yields of 28–64%. Aldehydes **33** were employed as the efficient dienophiles for the enantioselective Diels–Alder reaction.

### 2.2. α-Thio- and α-Seleniumsubstituted α,β-Unsaturated Aldehydes

The carbon–sulfur (C–S) bond is a key structural motif that is ubiquitous in bioactive compounds and pharmaceuticals [47,48,49,50].

**Scheme 12:**
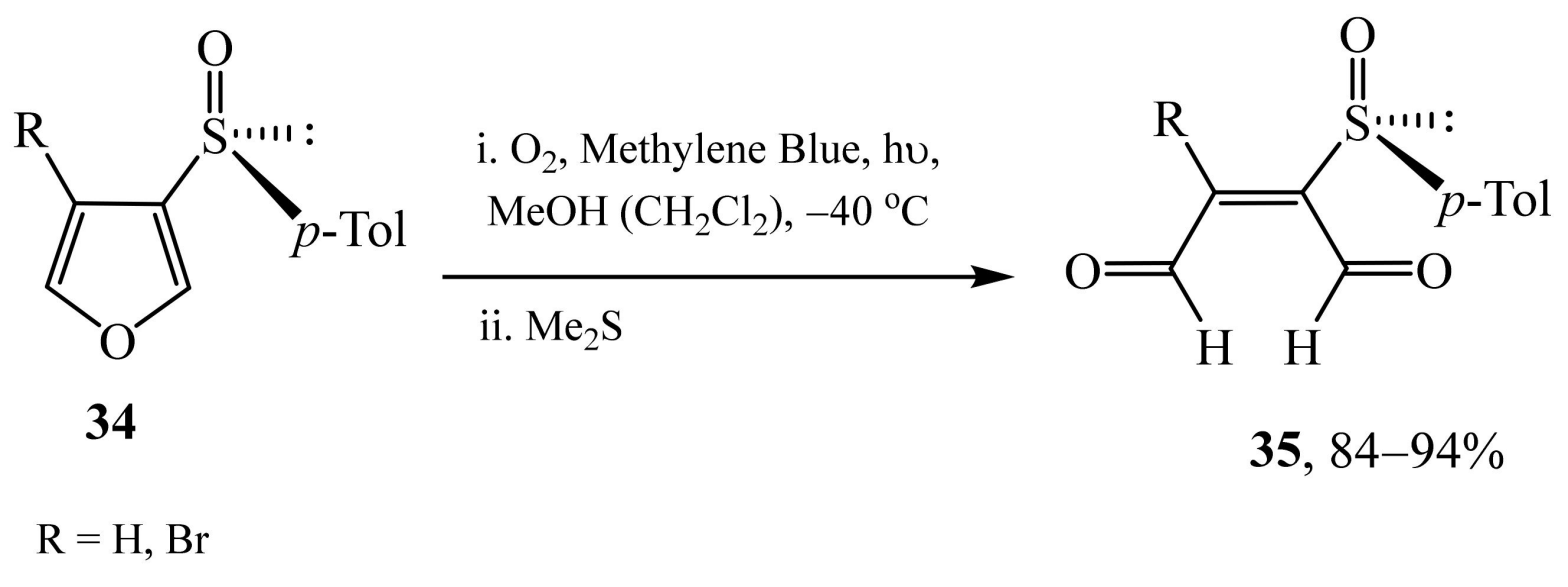
Synthesis of α-thiosubstituted α,β-unsaturated aldehydes **35** by ring opening of (*S*)-*p*-tolylsulfinylfurans **34**.

At the beginning of the 2000s, ring-opening reactions leading to the synthesis of α-thio-α,β-unsaturated aldehydes were implemented [51]. The photo-oxidized ring-opening of enantiomerically pure (*S*)-*p*-tolylsulfinylfurans in the presence of methylene blue yielded (*S*)-1,4-dicarbonyl-2-(*p*-tolylsulfinyl)-2-alkenes **35** (Scheme 12).

**Scheme 13:**
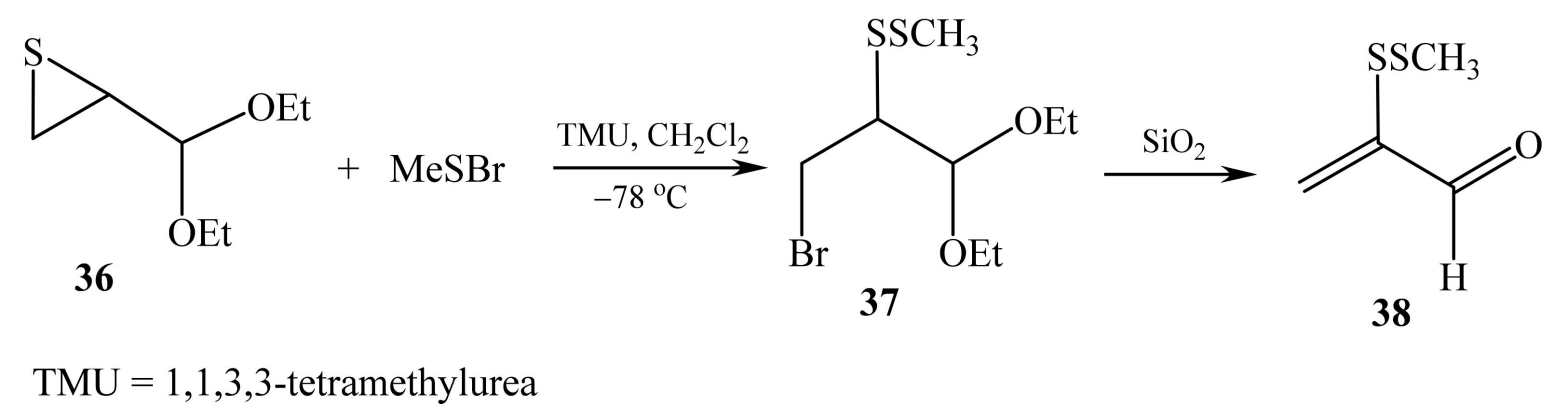
Synthesis of 2-disulfidesubstituted 2-propenal **38** by the ring-opening reactions.

Another ring-opening reaction was carried out by Silvestri and Wong [52]. It was shown that the thiirane cycle **36** was efficiently opened upon treatment with methanesulphenylbromide at low temperature in CH_2_Cl_2_ in the presence of 1,1,3,3-tetramethylurea (TMU). The formed halo disulfide **37** on silica gel produced predominantly aldehyde **38** via hydrolysis and dehydrohalogenation (Scheme 13).

Unfortunately, the yield of aldehyde **38** has not been reported.

**Scheme 14:**
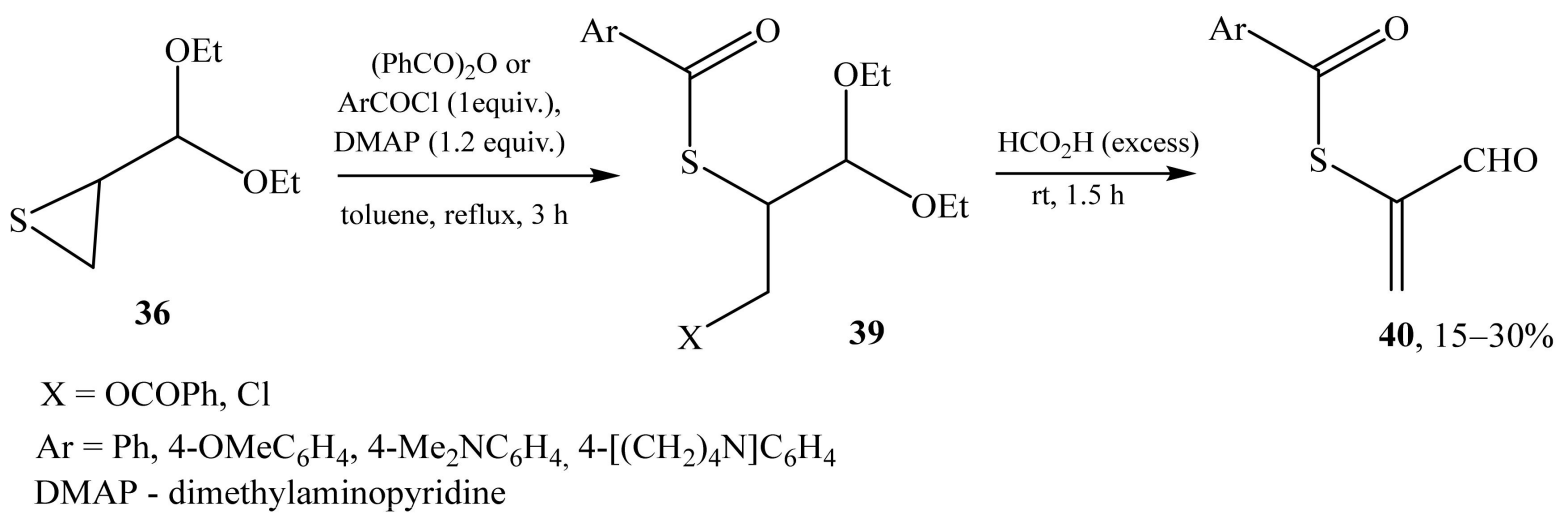
Synthesis of α-(benzoylthio)acroleins **40**.

In 2012, Ishihara et al. [37] first synthesized α-(benzoylthio)acroleins **40** by the acylation of thiirane **36** (Scheme 14).

The acylation of 2-(diethoxymethyl)thiirane (**36**) with benzoic anhydride or carboxylic chloride, followed by the hydrolysis with formic acid gave the corresponding α-(benzoylthio)substituted α,β-unsaturated aldehydes **40**. The modest yields of **40** were mainly attributed to the low reactivity of the thiirane in the acylation.

**Scheme 15:**
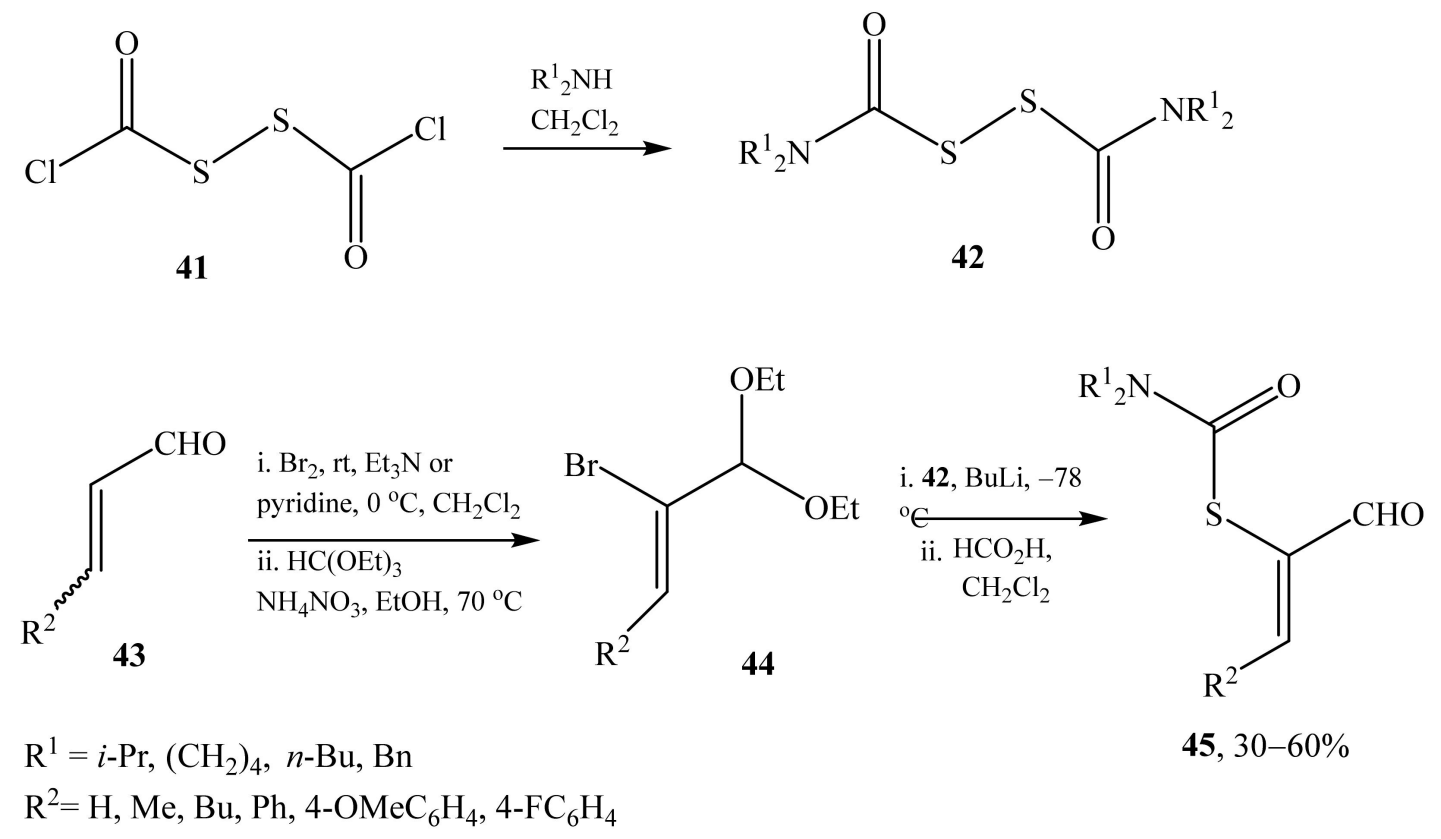
Synthesis of α-(carbamoylthio)substituted α,β-unsaturated aldehydes **45**.

α-(Benzoylthio)substituted α,β-unsaturated aldehydes **40** were synthesized and involved in Diels–Alder reactions as dienophiles to produce sulfur-containing quaternary carbons. However, the low basicity and poor solubility of aldehydes **40** did not allow the reaction with the diene to be performed. In this regard, a new synthetic approach to other promising dienophiles, α-(carbamoylthio)acroleins **45**, was developed. The approach was based on the umpolung strategy: the C–S bond formation between a “carbamoylthio cation R_2_NCO*S*^+^” and a “vinyl anion RCH=*CH^−^*” (Scheme 15).

Bis(carbamoyl)disulfides **42**, the synthetic equivalents of carbamoylthio cations, is prepared from bis(chlorocarbonyl)disulfide (**41**) and secondary amines. Lithiation of α-bromoacrolein diethylacetals **44**, obtained from aldehyde **43**, affords the corresponding vinyl anion. The vinyl anion with **42** followed by acid hydrolysis of the acetal moiety gives **45** in 30–60% yields. α-(Carbamoylthio)acroleins **45** shows high reactivity and enantioselectivity in the Diels–Alder reaction with 2,3-dimethylbutadiene.

**Scheme 16:**
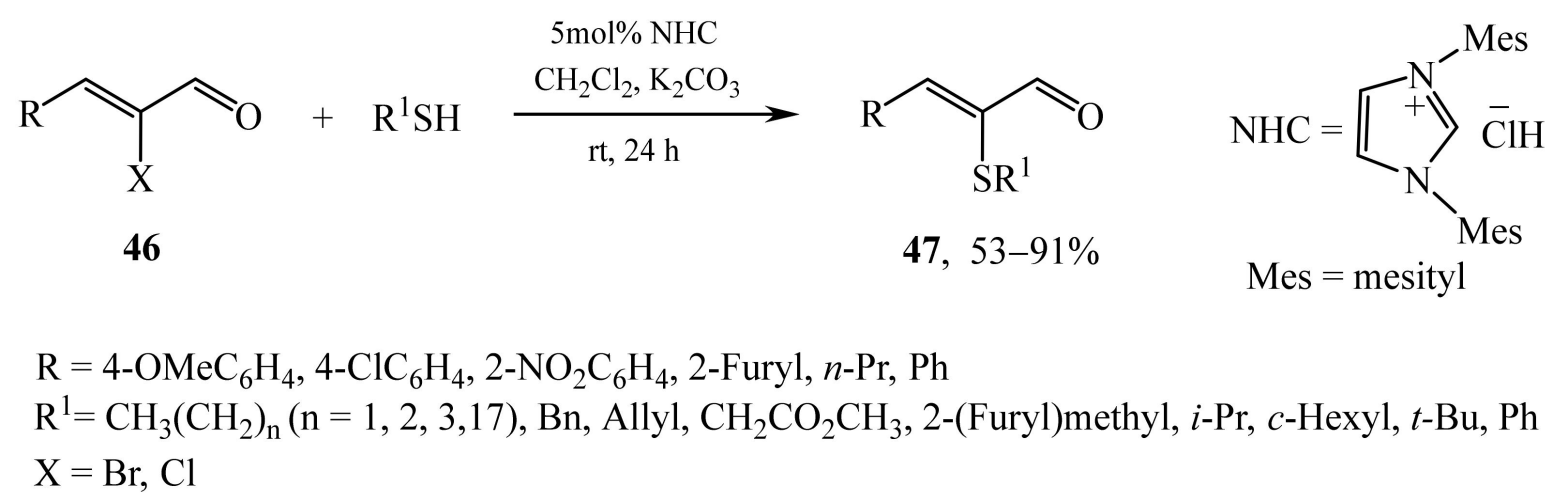
Synthesis of 2-sulfanylsubstituted 2-propenals **47** from α-haloenals **46**, thiols, and NHC.

In 2014, L. He [53] synthesized 2-sulfanylsubstituted 2-propenals via a new organocatalytic cross-coupling reaction between α-haloenals and thiols using NHC (N-heterocyclic carbene) as a catalyst (Scheme 16).

**Scheme 17:**
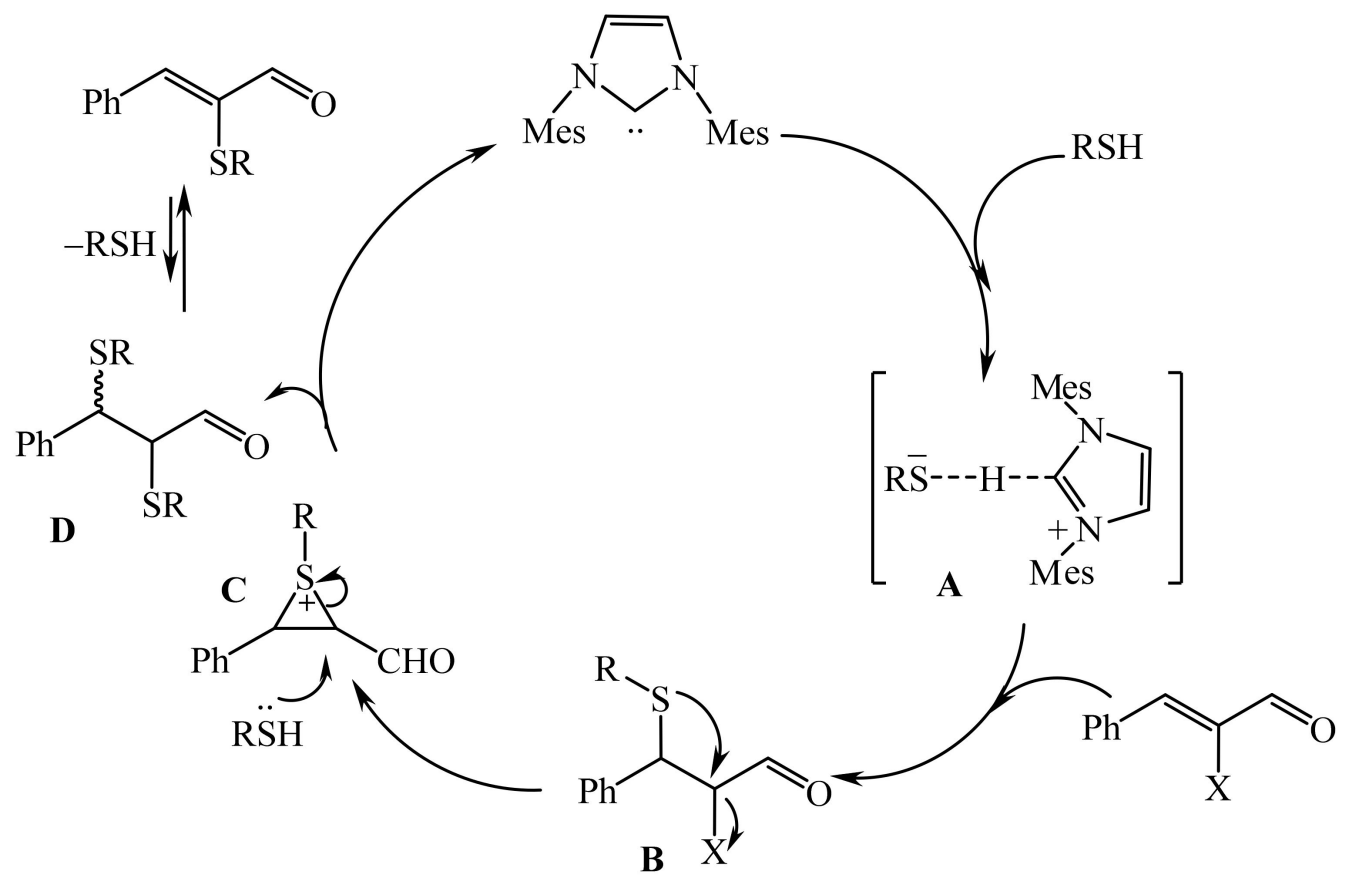
The proposed mechanism for formation of aldehydes **47**.

In the presence of 5 mol% NHC and potassium carbonate, various thiols reacted with α-haloenals **46** to afford 2-sulfanylsubstituted 2-propenals **47** in high yields (53–91%) and high *Z*-selectivity. Although the mechanism of this reaction is still obscure, it is likely based on the pioneering research of Movassaghi [54] and others [55,56,57] (Scheme 17).

In this process, NHC acts as a carbon-centered Brønsted base. The complex **A** (thioxy anion/azolium ion complex) is formed as a result of the deprotonation of the acidic thiol by NHCs. The complex **A** further interacts with α-haloenal to give compound **B**. The conjugate adduct **B** undergoes an intramolecular sulfenylation via a favorable 3-*exo-tert* attack to form sulfonium ion intermediate **C** according to Baldwin’s rule. The attack of the second thiol molecule leads to the ring-opening in the intermediate **C** to deliver bisulfenylated aldehyde **D**. Subsequent β-elimination produces α-alkylthiopropenal.

More readily available organocatalysts such as Et_3_N or DABCO can also be employed instead of N-heterocyclic carbene [58].

**Scheme 18:**
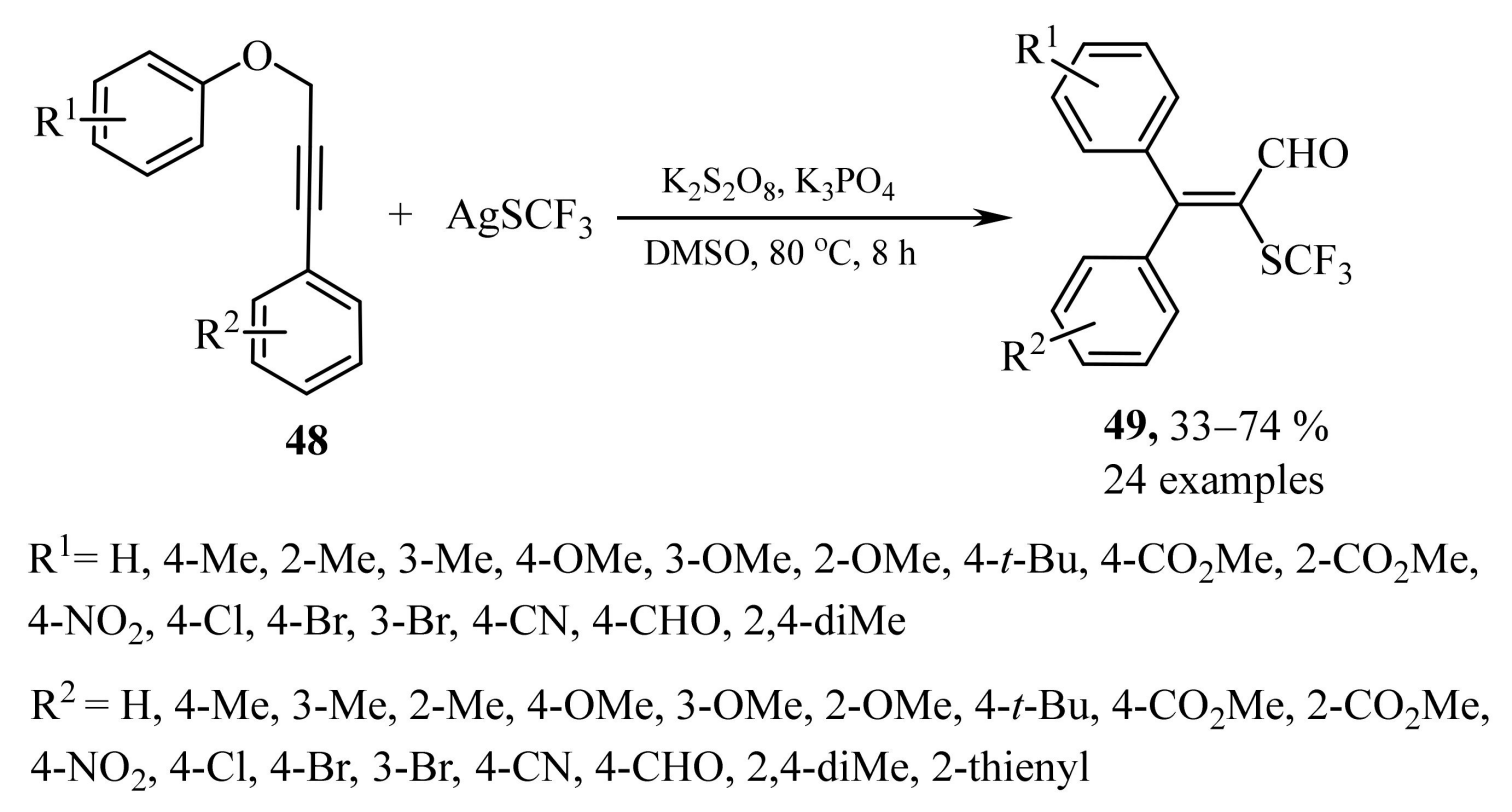
One-pot synthesis of 2-thiosubstituted 2-propenals **49**.

In 2017, Liu [59] carried out an original one-pot synthesis of 2-thiosubstituted 2-propenals **49** from 3-aryl-2-propynyl ethers **48** (Scheme 18). The method combines alkyne trifluoromethylthiolation, radical 1,4-aryl migration from oxygen to carbon and the formation of a carbonyl moiety.

**Scheme 19:**
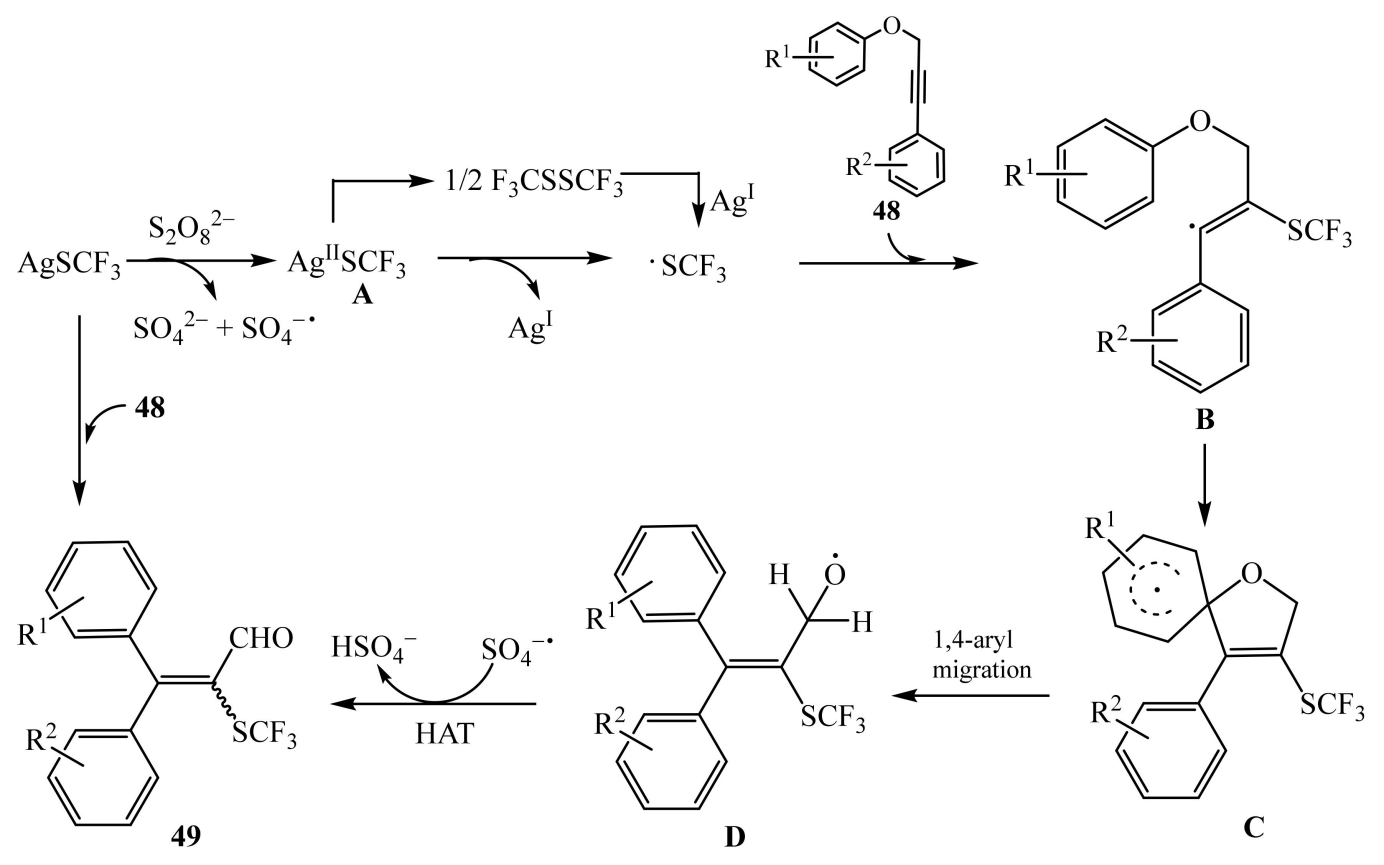
Proposed reaction mechanism.

The reaction features excellent conversion of aryl propynyl ethers into SCF_3_-containing α,β-unsaturated aldehydes through a radical pathway (Scheme 19).

At the first stage of the reaction, AgSCF_3_ is oxidized by K_2_S_2_O_8_ to give Ag^II^SCF_3_ (**A**), which could generate trifluoromethylthio radical (·SCF_3_) via single electron transfer. Further, this radical is regioselectively added to the triple bond of substrate **48**, affording a vinyl radical **B**. An intramolecular radical *ipso*-cyclization of **B** furnishes the spiro-intermediate **C**, and the subsequent 1,4-aryl migration leads to the intermediate **D**. The target product **49** is obtained via hydrogen atom abstraction (HAT) of radical intermediate species **D**.

A radical cascade reaction of the unsaturated C–C bonds involving migration is also of interest to the chemical community [60].

α-Seleniumcontaining α,β-unsaturated aldehydes are vinylmetallic reagents and valuable polyfunctionalized substrates [61,62]. Few methodologies to obtain 2-seleno-2-propenals were published [63,64,65,66].

**Scheme 20:**
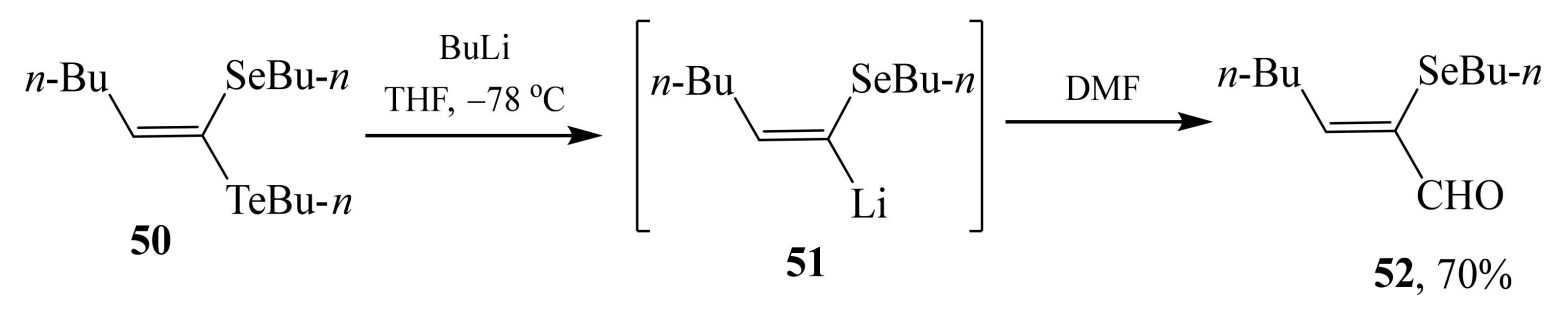
Synthesis of (*Z*)-2-(butylseleno)-2-heptenal (**52**).

In 2000, [67] α-(butylseleno)-α,*β*-unsaturated aldehyde (**52**) was synthesized in high yields from the lithiated vinyl selenide **51** generated in situ from ketene telluro(seleno)acetal **50** (Scheme 20). Aldehyde **52** has (*Z*)-configuration.

**Scheme 21:**
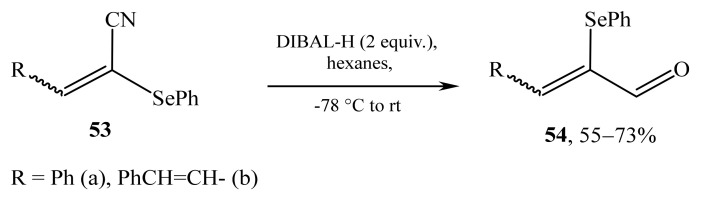
Synthesis of 2-phenylselenoalkenals **54**.

In 2001, Silveira et al. [68] reported a reduction in the CN moiety from α-phenylseleno acrylonitriles **53** in the presence of DIBAL-H (diisobutylaluminium hydride), giving rise to α-phenylselenosubstituted α,β-unsaturated aldehydes **54** (Scheme 21).

The α-phenylselenocinnamaldehyde (**54a**) is obtained as a mixture of the *Z* and *E* isomers in a ratio of 3.5:1. The α-phenylseleno-α,β,γ,δ-unsaturated aldehyde (**54b**) has the *Z*,*Z* configuration only.

### 2.3. α-Phenyl(aryl)substituted α,β-Unsaturated Aldehydes

2-Arylsubstituted 2-propenals represent valuable building blocks for organic synthesis [69,70]. The synthesis and properties of 2-phenylpropenal, usually called atropaldehyde, have received much attention in the medical literature, as this compound is known to be a cytotoxic metabolite of antiepileptic drug Felbamate [71,72].

**Scheme 22:**
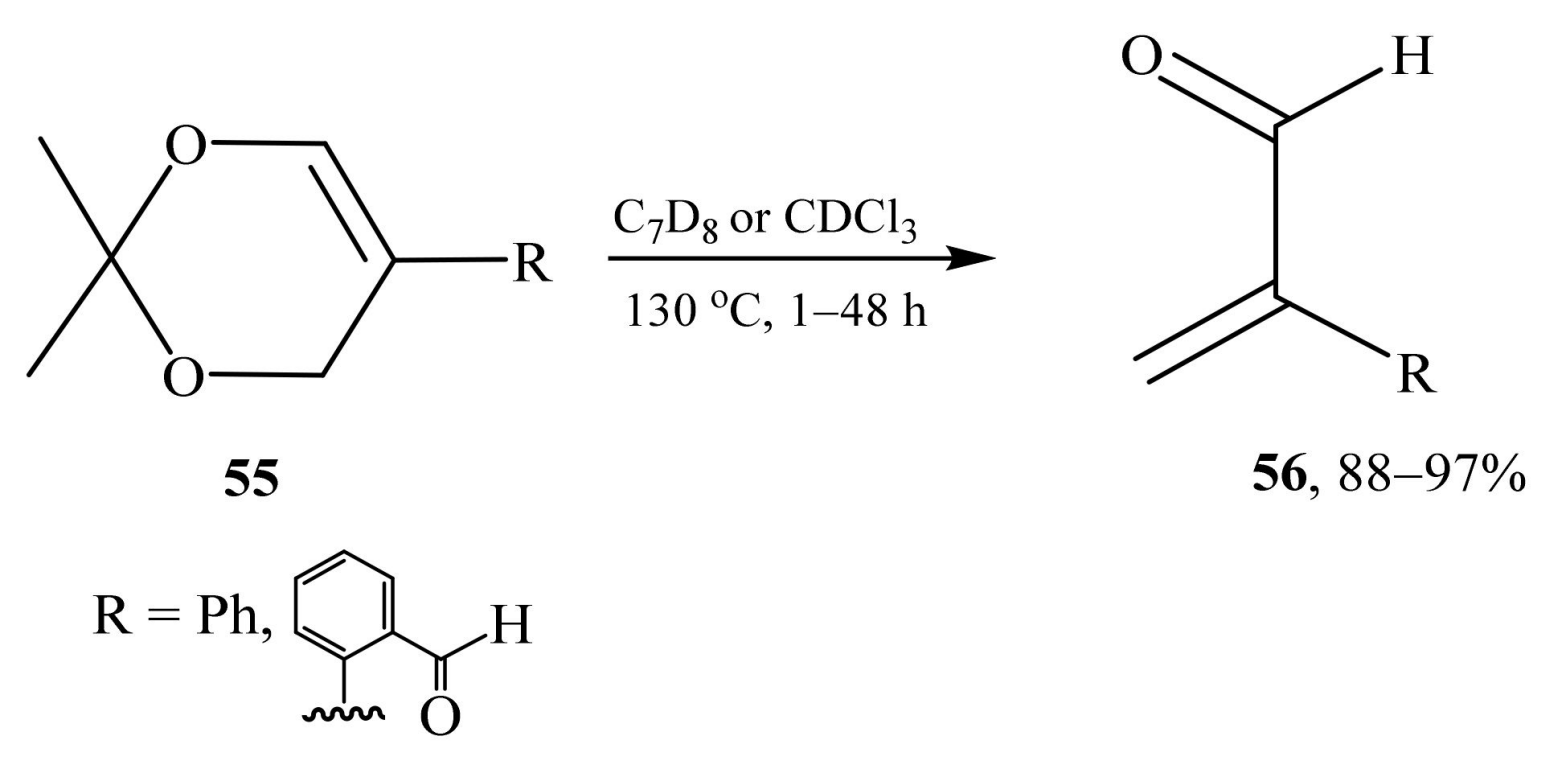
Thermolysis of 5-substituted-4*H*-1,3-dioxins **55**.

In 2000, an original method for the preparation of 2-phenylacroleins was developed by Funk et al. [73]. According to this method, 5-substituted-4*H*-1,3-dioxins **55** undergo smooth retrocycloaddition at 115–130 °C to the corresponding 2-phenylacroleins **56** (Scheme 22).

The retrocycloaddition reaction for each of dioxins **55** is performed without any diene or heterodienophilic trapping agent. Thus, the thermolyses of dioxins **55** cleanly gives the corresponding 2-substituted acroleins **56**. This method is of obvious interest, as it allows various α-substituted α,β-unsaturated aldehydes to be obtained [29,31].

**Scheme 23:**
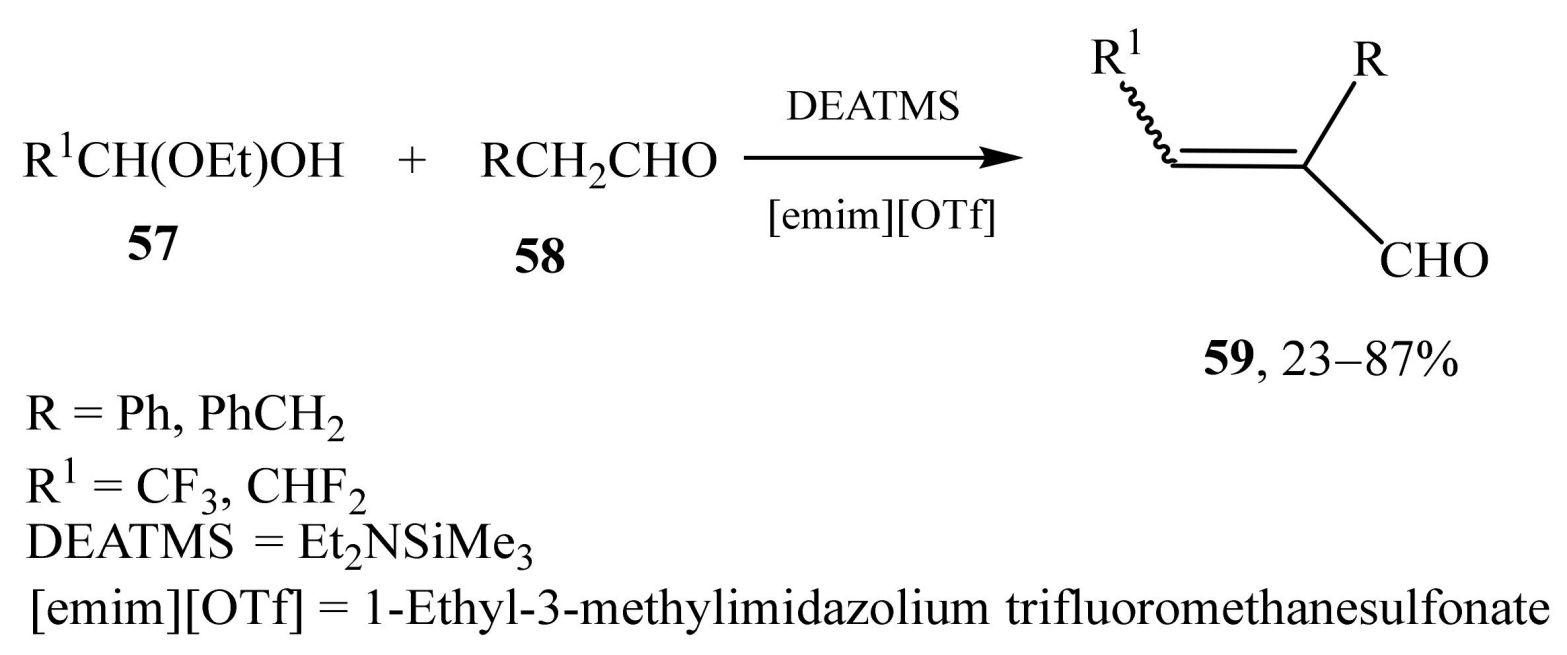
Synthesis of 3-fluoromethyl-2-substituted propenals **59**.

In 2004, Kitazume et al. [74] elaborated a one-stage synthesis of 2-substituted 3-tri-(or di-)-fluoromethyl-2-propenals using versatile aldehydes, tri- or di-fluoroacetaldehyde ethyl hemiacetal in the presence of diethylaminotrimethylsilane (DEATMS) in an ionic liquid. This protocol permits us to successfully construct 2-substituted propenals with high levels of selectivity of the geometric isomers (Scheme 23).

**Scheme 24:**
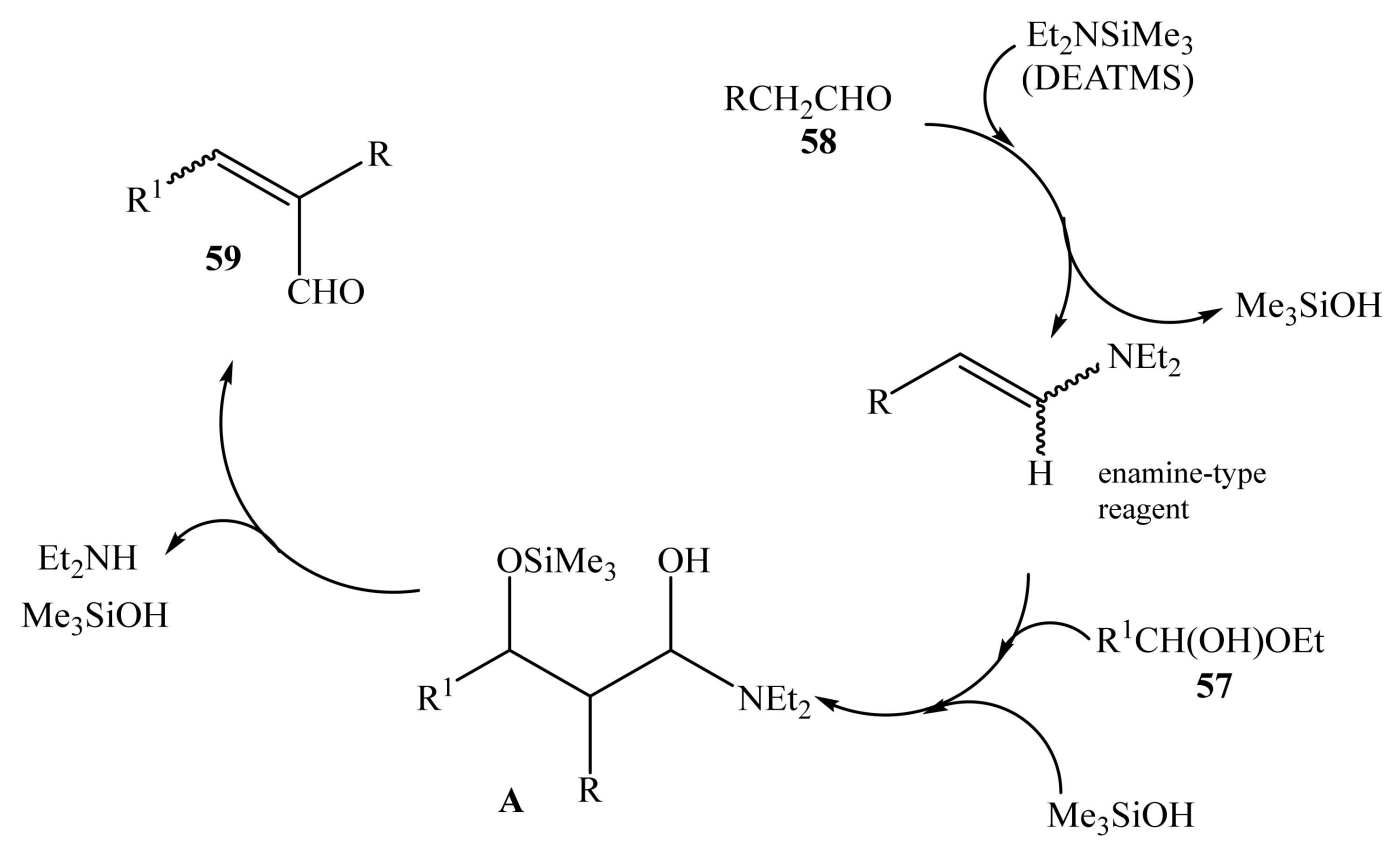
Proposed pathway to 2-propenals **59**.

The reaction of hemiacetal **57** with enamine, generated from DEATMS and aldehyde **58**, involves the generation of the intermediate **A** to produce 2-substituted 3-tri- (or di-) fluoromethyl-2-propenals **59** (Scheme 24).

Note that this reaction meets the requirements of green chemistry (solvent reuse), while reuse of the recovered ionic liquid in the same process ensured high yields of the product and good selectivity (like in the first cycle).

**Scheme 25:**
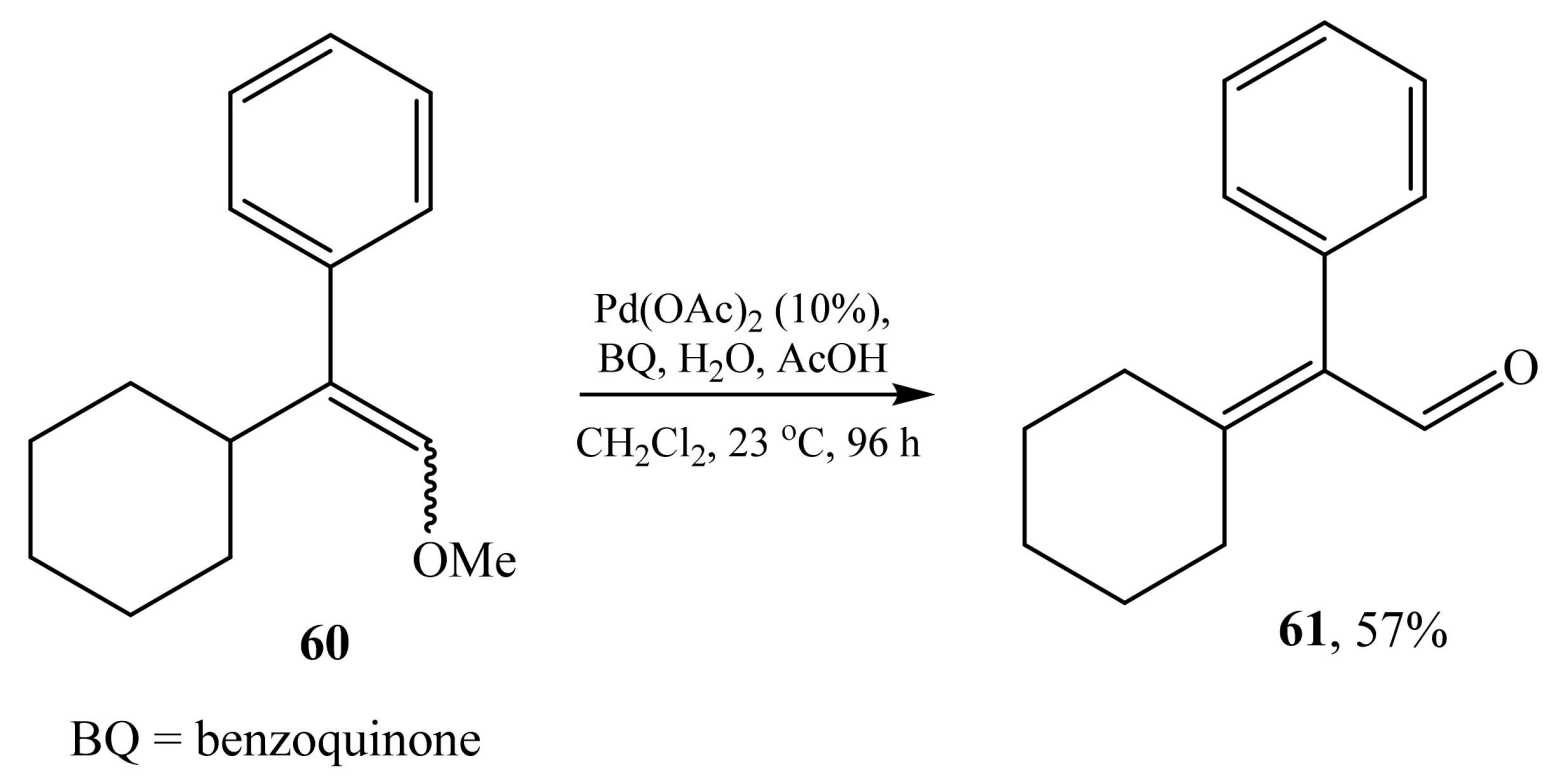
Synthesis of 2-cyclohexylidene-2-phenylacetaldehyde (**61**).

A wide series of α,β-unsaturated aldehydes including 2-cyclohexylidene-2-phenylacetaldehyde (**61**) was synthesized in 2012 by Stambuli et al. [75]. The mild oxidation of alkyl enol ether **60** takes place with the low loadings of a palladium catalyst (Scheme 25).

The mild oxidation conditions tolerate a diverse array of functional groups, thus allowing the formation of di-, tri-, and tetrasubtituted olefins.

More recently, Mura and coworkers developed a short-cut to α-substituted α,β-unsaturated aldehydes through the cross-dehydrogenative coupling of two different primary alcohols behaving as the latent aldehydes [76]. This approach represents a cascade reaction, wherein a nonenolizable aldehyde is first generated in situ by the removal of a “hydrogen molecule” from an alcohol, and then is temporarily trapped as an imine.

**Scheme 26:**
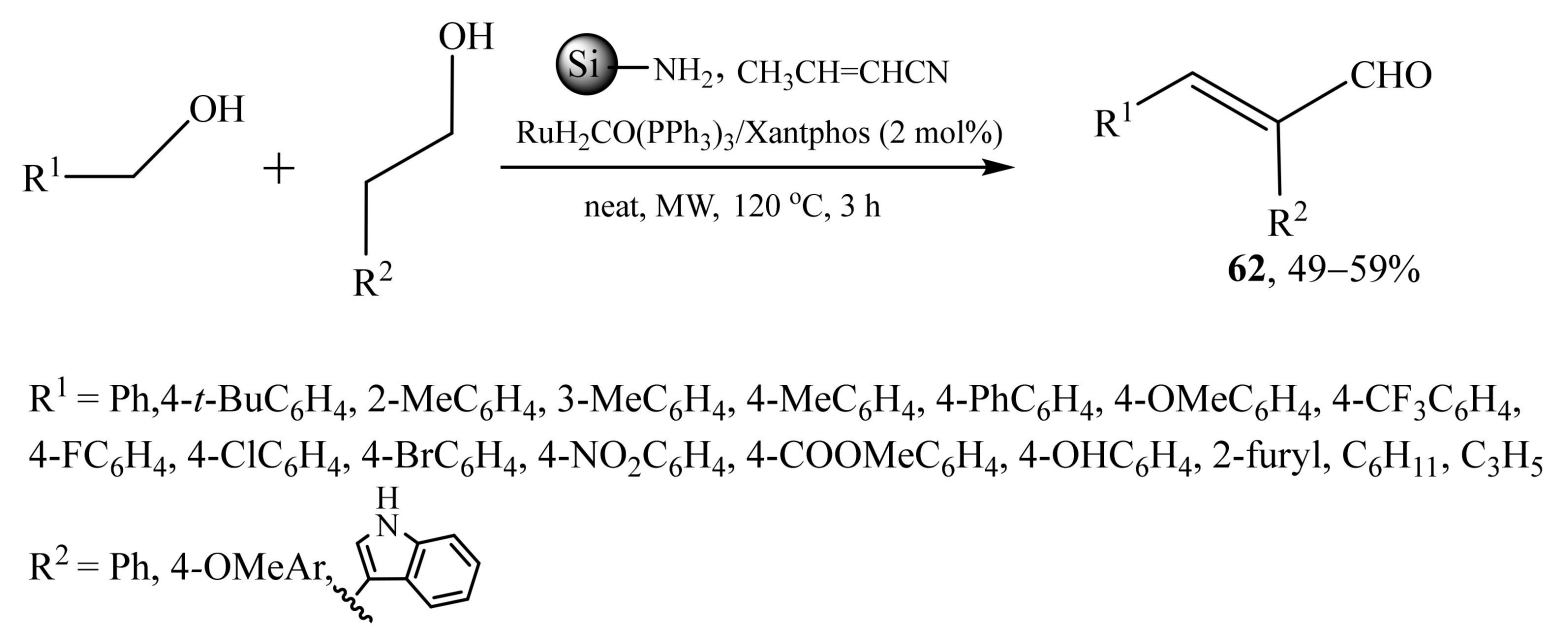
Synthesis of aldehydes **62** from alcohols.

The target 2-alkenals **62** are formed due to the subsequent Mannich-type condensation between the imine particles and another intermediate aldehyde (Scheme 26).

**Scheme 27:**
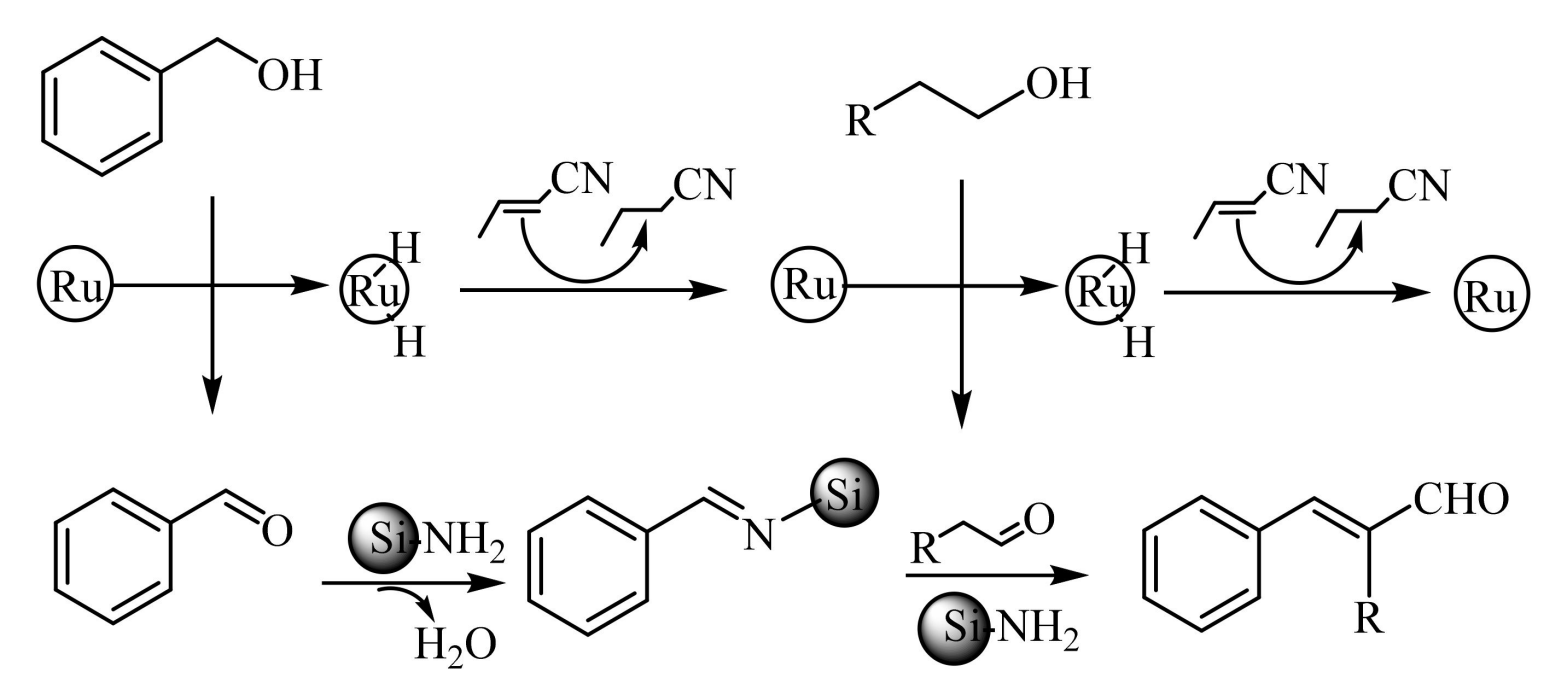
Plausible reaction mechanism.

A plausible mechanism of this ruthenium-promoted transfer-hydrogenation/Mannich-type domino reaction is shown in Scheme 27. Higher reactive benzyl alcohol is first oxidized by the ruthenium-mediated hydrogen transfer from the substrate to crotononitrile. Further, the formed benzaldehyde is probably trapped by the supported amine, leaving the restored catalyst to oxidize the aliphatic alcohol via a second hydrogen transfer with crotononitrile. Finally, the Mannich-type reaction between the grafted imine species and the second aldehyde delivers the α-substituted α,β-unsaturated aldehydes **62**.

The use of enolates prepared in situ from alcohols helps to avoid the treatment of unstable aldehydes, and opens the way to various cinnamic aldehydes bearing a substituent in the α-position. The addition of a silica-grafted primary amine leads to a selective one-pot process to produce cross-dehydrogenative coupling products in good-to-moderate yields and with high chemoselectivity.

### 2.4. α-Amino- and α-Amidosubstituted α,β-Unsaturated Aldehydes

2-Amino- and 2-acylamino-3-alkylpropenals are unique groups of organic compounds, the synthesis of which, however, still remains underdeveloped. Such compounds are known to be excellent dienophiles for Diels–Alder reactions and subsequent design of bioactive natural compounds [77,78]. The products of Diels–Alder cycloaddition reactions of 2-amidoacroleins with dienes are structural motifs of biologically active tricyclic alkaloids (cytotoxins lepadiformine, fasicularin, cylindricines A/B) [79,80,81,82] and immunosuppressent FR901483 [83].

**Scheme 28:**
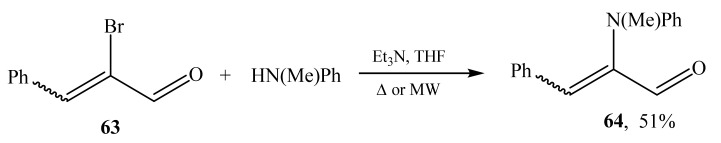
The formation of 2-amino-2-alkenal **64**.

In 2003, Rulev et al. [84] synthesized for the first time enamine **64** bearing a weakly basic tertiary amino group. (*Z*,*E*)-2-[Methyl(phenyl)amino]-3-phenylpropenal (**64**) was obtained by the reaction of 2-bromo-3-phenylalkenal **63** with *N*-methylaniline in the presence of Et_3_N in anhydrous THF. The reaction proceeds via the nucleophilic vinyl substitution of halogen atom in the activated haloalkene. This reaction takes 30 h at 90 °C. After purification on silica gel, the yield of 2-amino-3-phenylpropenal **64** is 51% (Scheme 28).

**Scheme 29:**
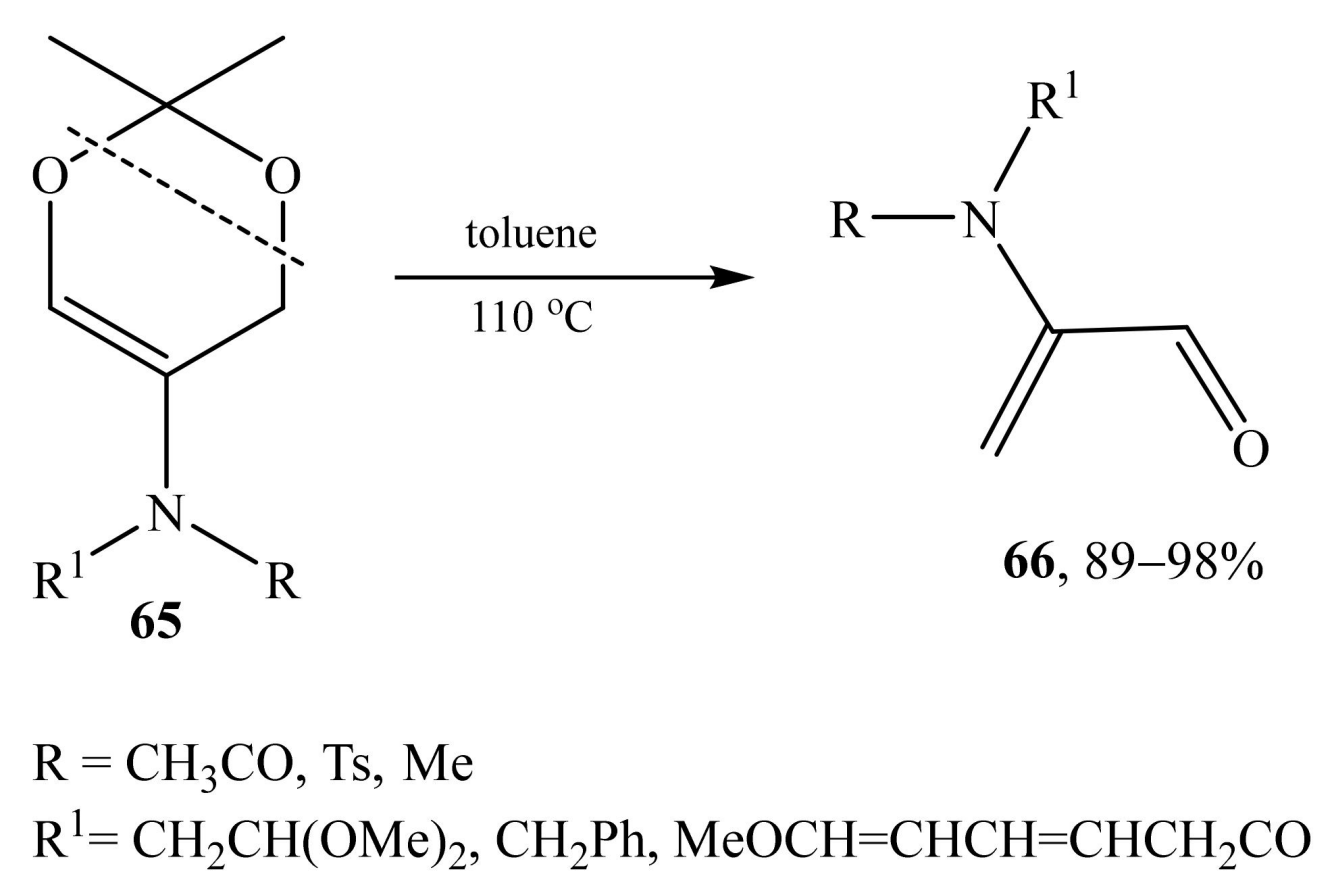
The synthesis of 2-amido-2-alkenals **66** by retrocycloaddition of 5-amido-1,3-dioxin.

Various 2-amido-2-alkenals **66** were synthesized by the retrocycloaddition of 5-amido-1,3-dioxin **65** in refluxing toluene [79] (Scheme 29).

**Scheme 30:**
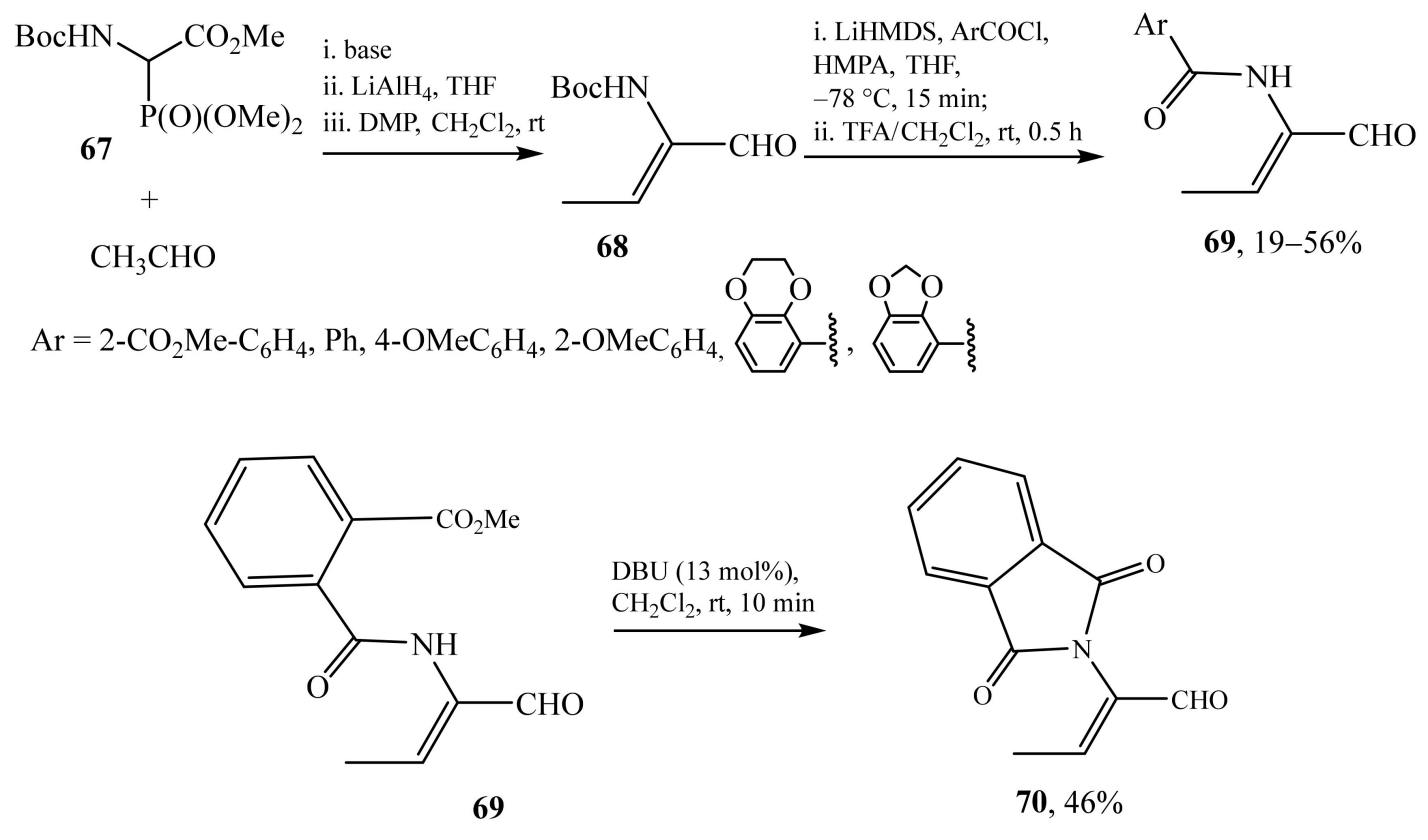
Synthesis of α-nitrogensubstituted α,β-unsaturated aldehydes **69** and **70**.

In 2012 [46], various α-nitrogensubstituted α,β-unsaturated aldehydes **68** were prepared from aldehydes and 2-acylamino-2-(dimethoxyphosphoryl)acetate **67** by the method developed by Burk et al. [85] (Scheme 30).

The subsequent removal of the tert-butyloxycarbonyl group (Boc) from aldehyde **68** gives 2-(acylamino)crotonaldehydes **69** in 19–56% yields. Additionally, the treatment of crotonaldehyde **69** with DBU (13 mol%) produces highly reactive 2-phthalimidoacrolein (**70**) in 46% yield.

**Scheme 31:**
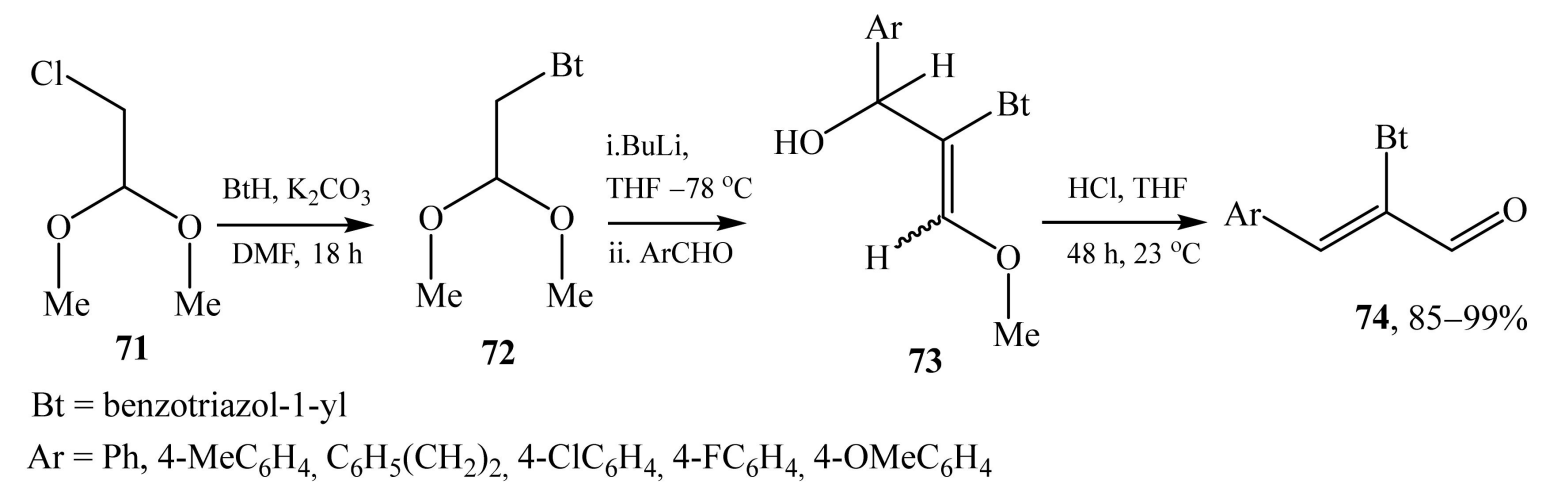
Synthesis of α-benzotriazolyl-α,β-unsaturated aldehydes.

Three-stage method for the synthesis of α-benzotriazolyl-α,β-unsaturated aldehydes **74** (Scheme 31) was disclosed by Katritzky et al. [86].

2-Chloroacetaldehyde dimethylacetal (**71**) reacts with benzotriazole in DMF to give 1-(2,2-dimethoxyethyl)-1H-1,2,3-benzotriazole **72**. The acetal carbanion **72** formed under the action of *n*-BuLi attacks the carbonyl group of the aromatic aldehyde (ArCHO) to result in vinylbenzotriazoles **73**, the hydrolysis of which affords 2-triazolylcinnamaldehydes **74** in high yields. Aldehydes **74** are used as synthons in the pyrazole synthesis.

In 2006 [24], a series of α-substituted acroleins (including α-NHBoc-2-alkenals) was obtained by mild α-methylenation of aldehydes in the presence of 10 mol% pyrrolidine/propionic acidic or 10 mol% L-pro-β-Ala. The reaction proceeds chemoselectively for 1–4 h at 45 °C to ensure 64–99% yields of the functionalized α-substituted acroleins. A year later, the authors optimized the reaction conditions using a combination of pyrrolidine and a weak acid co-catalyst (*p*-dimethylaminobenzoic acid) [25] that significantly shortened the reaction time. For instance, 2-aminosubstituted aldehyde is prepared for 15 min in 98% yield. Formerly, similar 2-substituted aldehydes **16**, **17** are synthesized by the above methodology (Scheme 4, Section 2.1).

### 2.5. α-Halosubstituted α,β-Unsaturated Aldehydes

Halovinyl aldehydes hold a prominent place in organic chemistry as valuable building blocks for the targeted design of linear and heterocyclic systems [87]. Therefore, the development of new methods for the preparation of these compounds remains an urgent challenge.

Most often, α-chloro- and α-bromosubstituted α,β-unsaturated aldehydes are obtained via halogenation of the corresponding α,β-unsaturated aldehydes followed by dehydrohalogenation. Di- and triethylamine are used as HHal acceptors. However, yields of the target products reach only 36–68% [88,89]. On the other hand, it was shown that bromination/dehydrobromination of *E*-hexenal at 0–5 °C afforded (*Z*)-2-bromohex-2-enal in a quantitative yield [90].

**Scheme 32:**
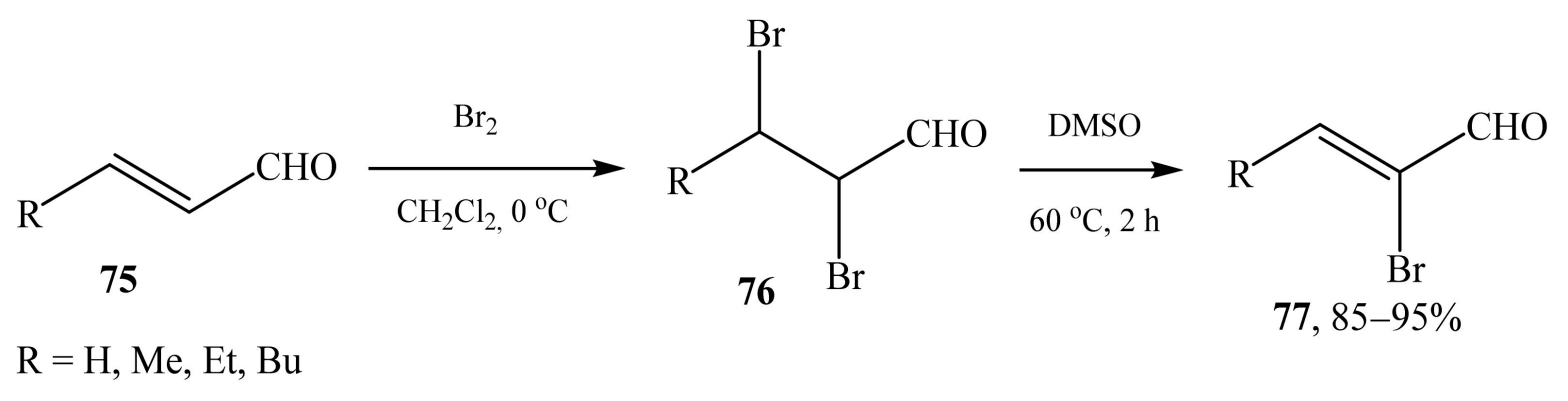
Formation of 2-bromopropenals **77**.

In 2007, Li et al. [91] obtained 2-bromopropenals **77** by bromination of α,β-unsaturated aldehydes **75** in the presence of dimethyl sulfoxide, which induced effective selective dehydrohalogenation of α,β-dihalo compounds **76** (Scheme 32).

The two-stage reaction proceeds without alkalis under mild conditions. The process is facile and allows analogs of 2-bromopropenals to be prepared in high yields. The DMSO acts in the reaction as a reagent and a solvent. Among the existing protocols of dehydrohalogenation, this method appears to be the most expedient and environmentally benign.

**Scheme 33:**
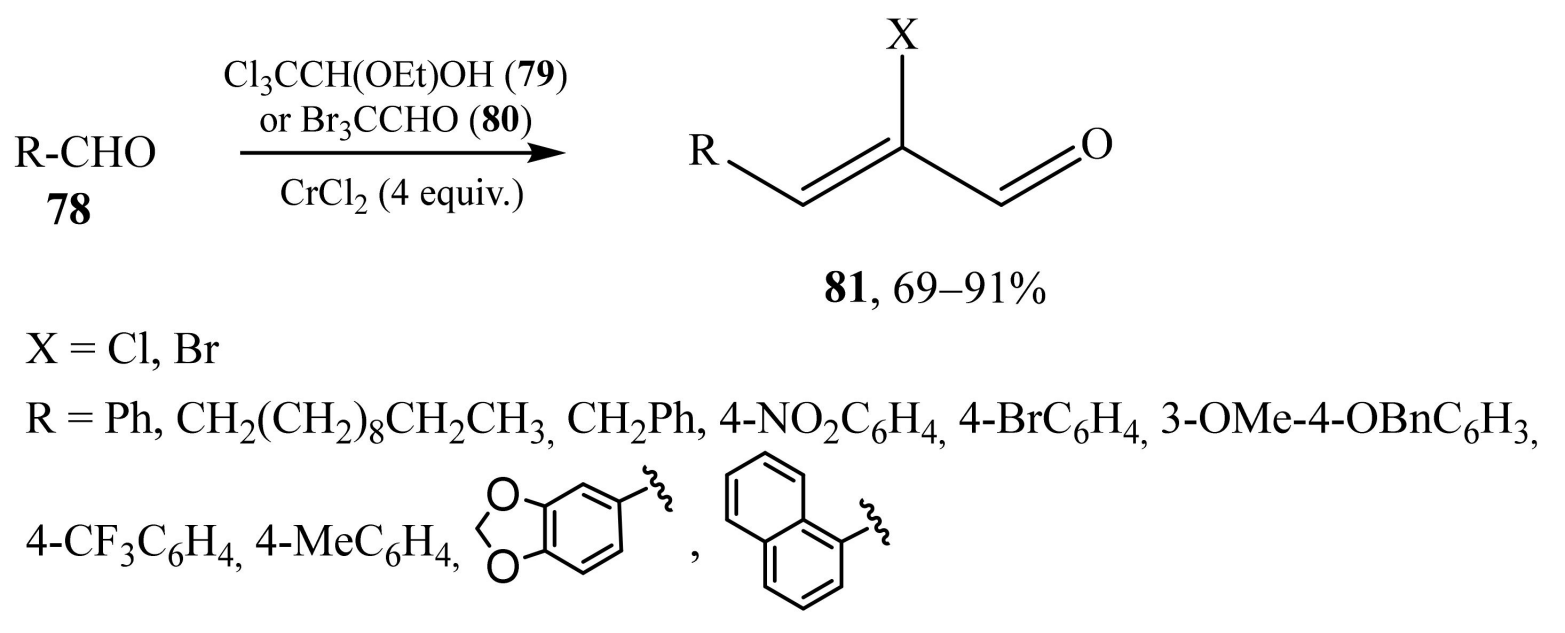
The CrCl_2_-mediated two-carbon halo-homologation of aldehydes **78**.

The CrCl_2_-mediated two-carbon halo-homologation of aryl, alkenyl, and aliphatic aldehydes **78** with chloral ethyl hemiacetal **79** or bromal **80** furnishes (*Z*)-α-chloro- and (*Z*)-α-bromo-α,β-unsaturated aldehydes **81** in good yields and high stereoselectivity [92] (Scheme 33).

**Scheme 34:**
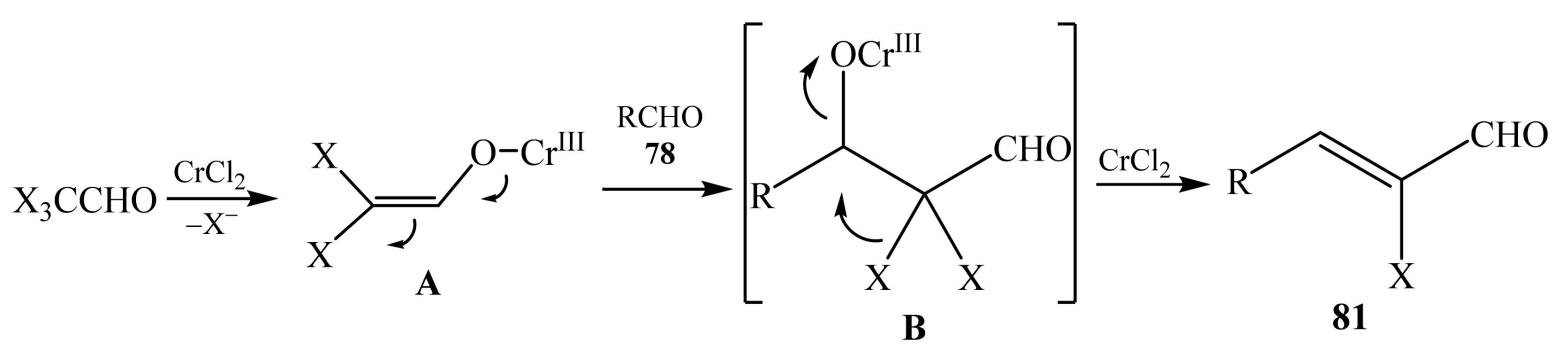
A tentative mechanism of the reaction halo-homologation of aldehydes.

According to the reaction mechanism, CrCl_2_ as a multiple one-electron reductant generates chromium(III)-enolate **A** from chloral/bromal, which is intercepted by the aldehyde (Scheme 34). The Reformatsky-type adduct **B** further undergoes the reductive metalation with concomitant E_2_-elimination to give the target α-Cl(Br)propenal **81**.

The method is applicable to the synthesis of (*Z*)-2-chloropentadec-2-enal, a toxin isolated from the marine red alga Laurencia *flexilis*. It is important that the reaction tolerates a variety of functional groups.

**Scheme 35:**
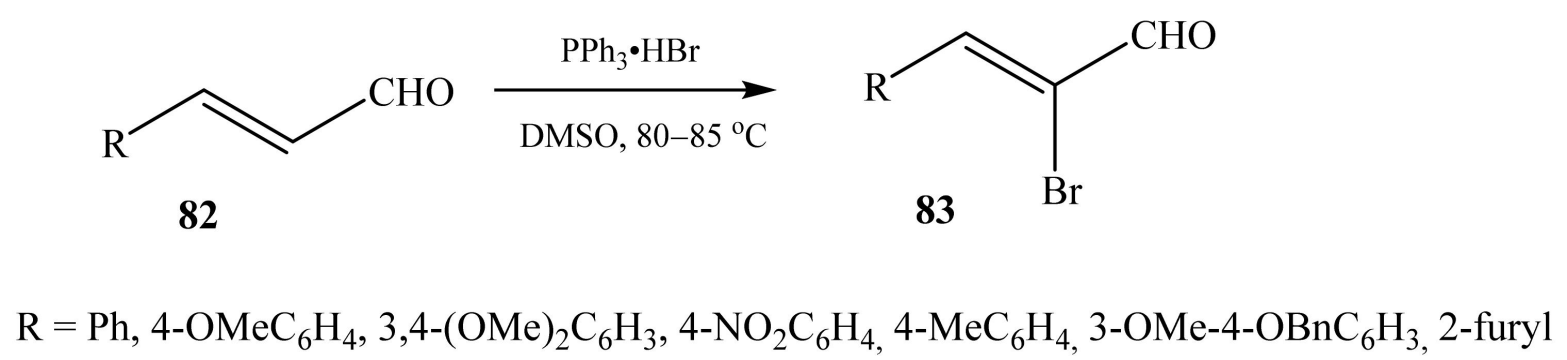
Synthesis of 3-aryl(hetaryl)substituted 2-bromopropenals **83** from enals **82**.

Das et al. [93] successfully elaborated an efficient chemoselective general procedure for the synthesis of 3-aryl(hetaryl)substituted 2-bromopropenals **83** from enals **82** through an unprecedented PPh_3_·HBr-DMSO mediated oxidative bromination followed by the Kornblum oxidation, yields of the products being from moderate to good (Scheme 35).

The mechanism of the transformation likely involves the initial oxidative dibromination of the olefinic double bond, followed by substitution of the bromine atom at the β-position by DMSO to generate alkoxysulfonium intermediate. Finally, elimination of the hydrogen atom leads to dehydrobromination. Alternatively, the intermediate bromonium derivative eliminates a proton from the α-carbon atom to directly give the product. The synthesis of such aromatic α-bromoenal derivatives generally comprises two steps, requires highly corrosive and toxic Br_2_, followed by base treatment. Therefore, one-stage protocol using the system PPh_3_•HBr-DMSO is an efficient alternative to the existing methods.

It is well known that the introduction of fluorine atoms into organic molecules often drastically changes chemical properties and biological activity of the parent compounds [94]. Over the last decade, fluorinated compounds attract noticeable interest. Due to the unique combination of physical-chemical and biological properties, achieved by incorporation of fluorine or perfluoroalkyl groups (usually CF_3_) in an organic molecule, fluorinated compounds find widespread application in design of new materials, agrochemicals, and drugs [95].

**Scheme 36:**
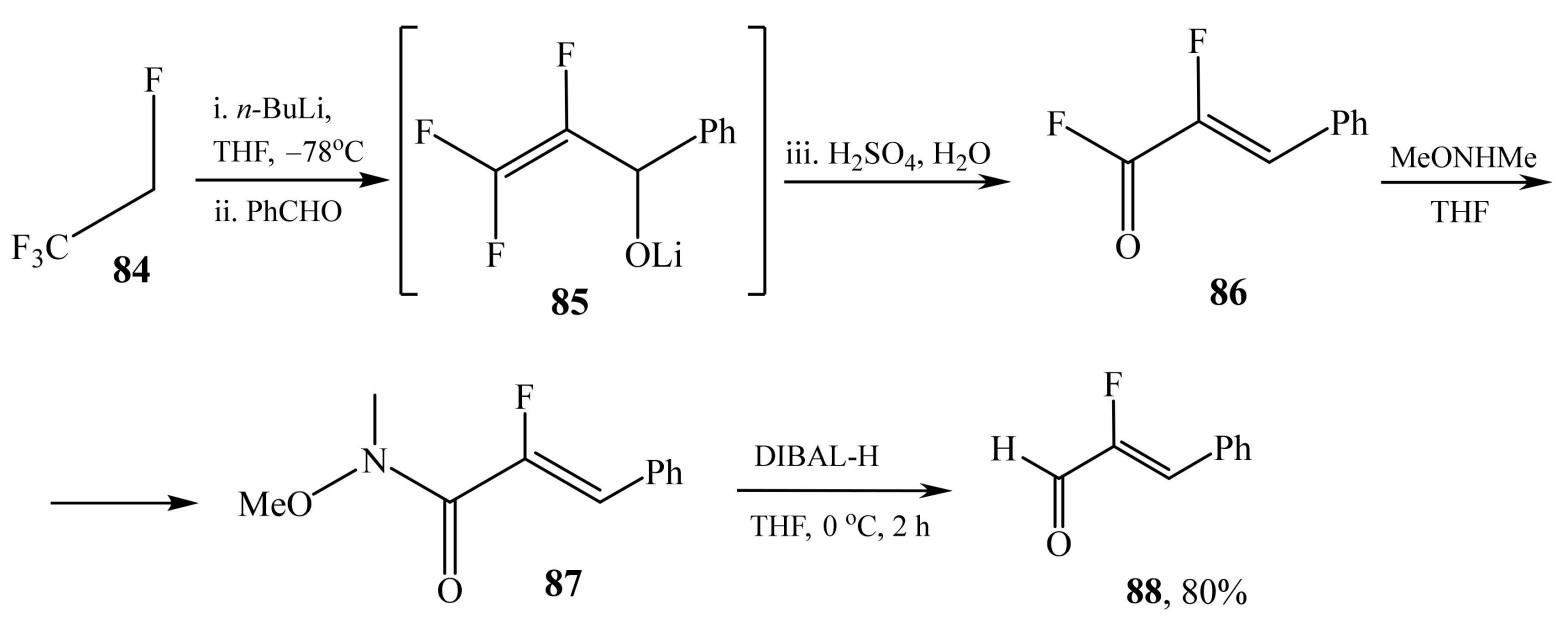
Synthesis of 3-phenyl-2-fluoro-2-propenal **88** from HFC-134a (**84**).

α-Fluorosubstituted α,β-unsaturated aldehydes has been synthesized by the reduction in 2-halogenated nitrosyl and ester functional groups with DIBAL-H in THF/Et_2_O to give aldehydes. Kanai et al. [96] affected stereoselective synthesis of 3-phenyl-2-fluoro-2-propenal (**88**) from the available 1,1,1,2-tetrafluoroethane (HFC-134a) (**84**) (Scheme 36).

The reactions sequence includes dehydrofluorination/metallation of **84** upon treatment with *n*-butyllithium in THF. Then, the organolithium adduct is added to the carbonyl group of benzaldehyde. Hydrolysis of **85** by H_2_SO_4_ gives acyl fluoride **86**, which is aminated with methoxymethylamine to deliver enamide **87**. The latter is reduced with diisobutylaluminum hydride (DIBAL-H) to produce 3-phenyl-2-fluoro-2-propenal (**88**) in high yield.

The obvious benefit of the process is that it proceeds as a two-pot sequence, without isolation of the intermediate products and with formation of only one *Z*-isomer.

**Scheme 37:**
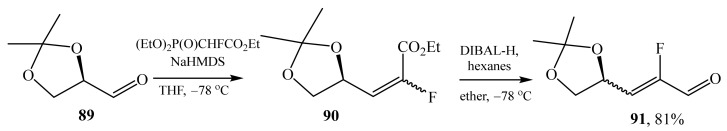
Synthesis of α-fluoroenal **91** from 1,2-*O*-isopropylidene-D-glyceraldehyde.

In 2002, Chu et al. [97] developed a method for the synthesis of original α-fluoroenal **91** from 1,2-*O*-isopropylidene-D-glyceraldehyde (**89**) (Scheme 37).

1,2-*O*-Isopropylidene-D-glyceraldehyde (**89**) reacted with diethyl α-fluorophosphonoacetate to give a mixture of fluoroesters in a 9:1 ratio, the (*E*)-isomer being predominant. The treatment of **90** with DIBAL-H/hexanes affords α-fluorosubstituted α,β-unsaturated aldehyde **91** as a mixture of the (*E*)- and (*Z*)-isomers. The isomers are separated by flash silica gel column chromatography. The (*E*)-3-[(S)-2,2-dimethyl-(1,3)-dioxolan-4-yl]-2-fluoro-propenal (**91**) is used as a synthon in preparation of D- and L -series of 3ʹ-fluorosubstituted pyranosyl nucleosides.

**Scheme 38:**
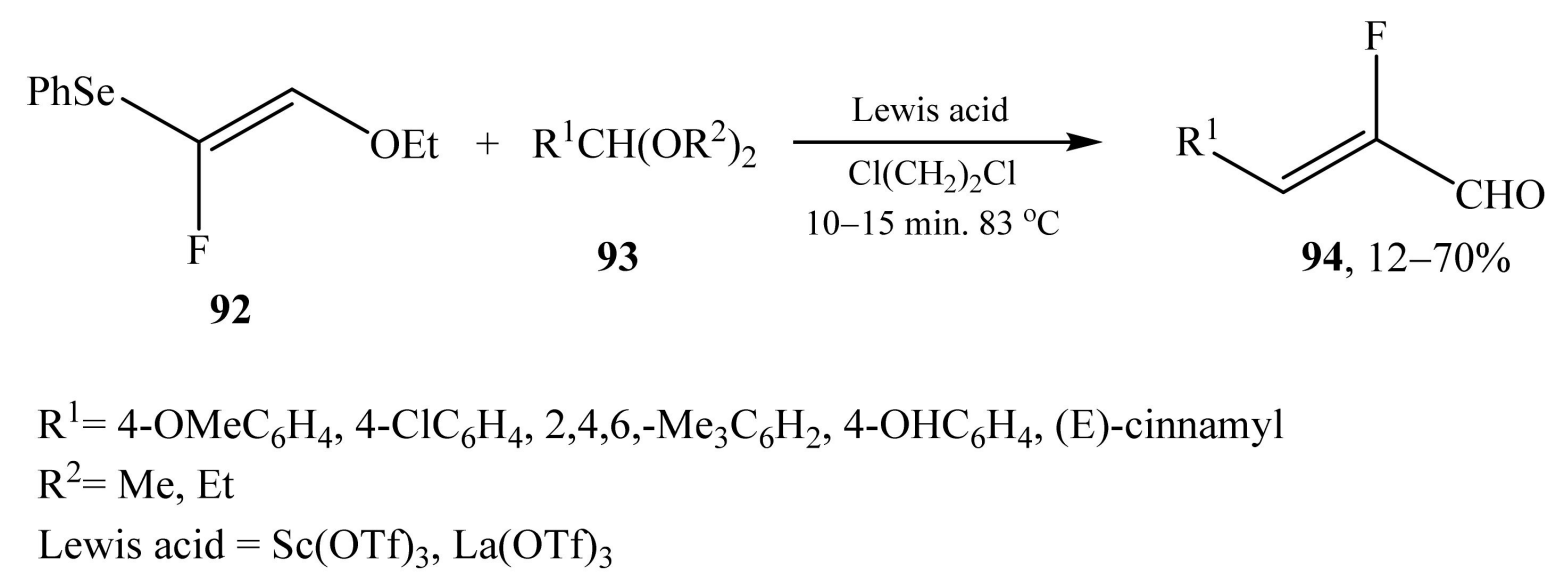
Lewis acid-catalyzed α-fluoroformylalkenylation of aldehyde acetals.

Later, Yoshimatsu and coworkers [98] synthesized 2-fluoroalkenals **94** from β-ethoxy-α-fluoro-α-(phenylselanyl)ethene (**92**) specially prepared by a multistage procedure (Scheme 38).

Ethene **92** is successfully utilized in the Lewis acid-catalyzed α-fluoroformylalkenylation of non-enolizable aldehyde acetals **93** to provide α,β-unsaturated aldehydes **94** in moderate yields.

**Scheme 39:**
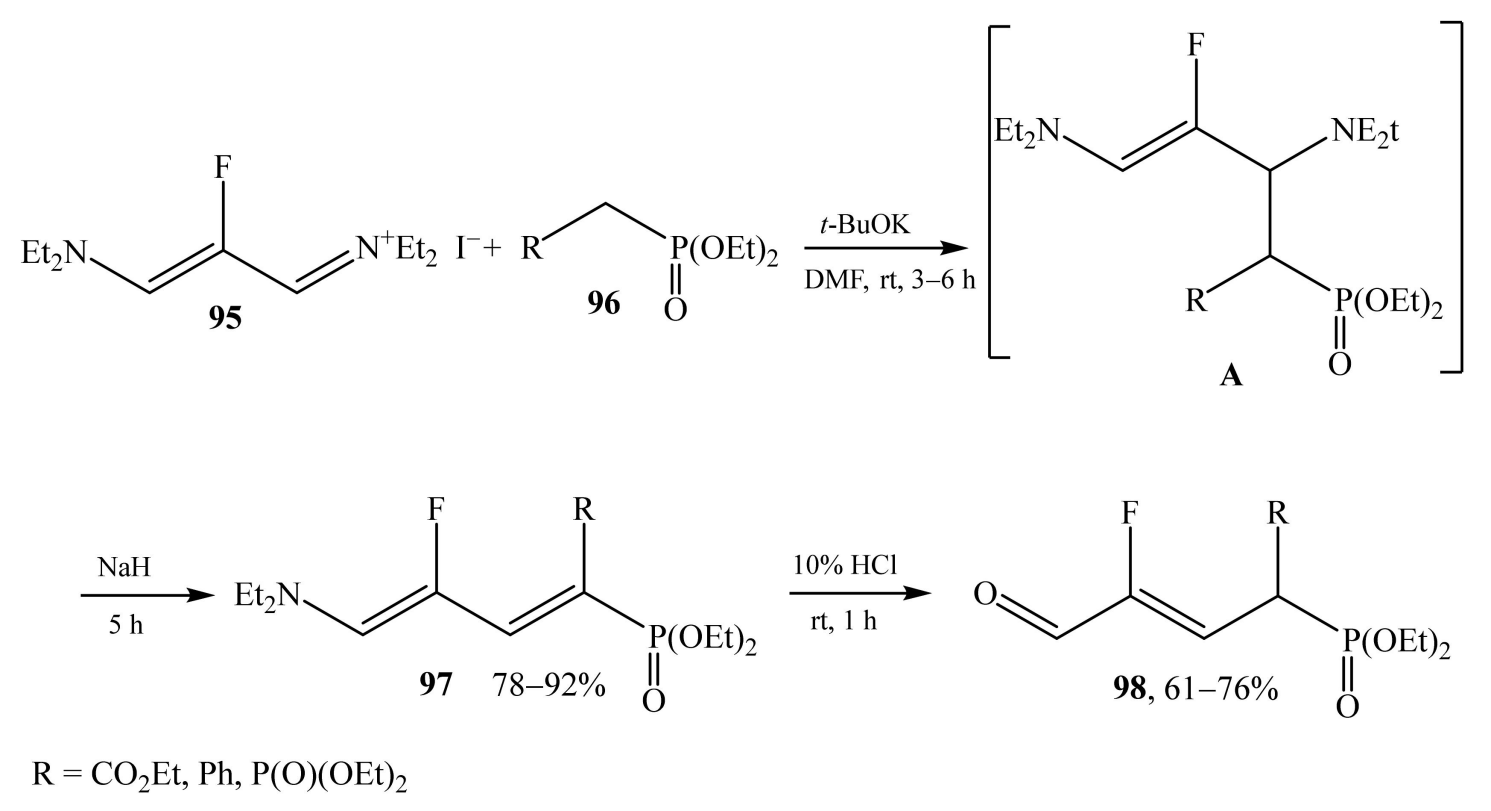
Synthesis of phosphonate-containing α-fluorinated α,β-unsaturated aldehydes **98**.

The reaction of β-fluorovinamidinium salt **95** with the Horner–Wadsworth–Emmons reagents **96** proceeds under basic conditions to furnish fluorinated 1,3-butadienyl phosphonates **97**. The substrate **97** is hydrolyzed with a 10% HCl aqueous solution to afford the corresponding γ-(diethylphosphono)-α-fluoro-α,β-unsaturated aldehydes **98** in good yields (Scheme 39) [99].

**Scheme 40:**
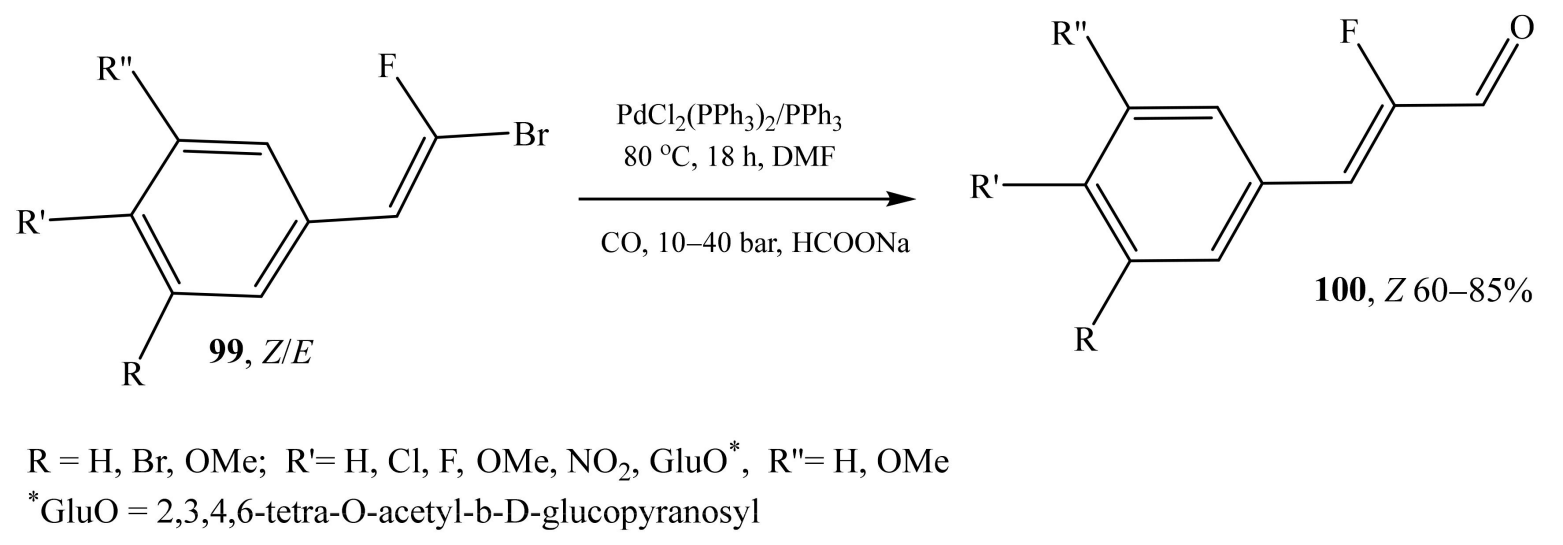
PdCl_2_(PPh_3_)_2_-catalyzed reaction of β-bromo-β-fluorostyrene **99** with CO and HCOONa.

The stereoconvergent Pd-catalyzed formylation of (*E*/*Z*)-β-bromo-β-fluorostyrene mixtures with carbon monoxide and sodium formate was implemented. Under the optimized conditions, the corresponding pure (*Z*)-α-fluorocinnamaldehydes **100** are obtained in good yields [100] (Scheme 40).

**Scheme 41:**
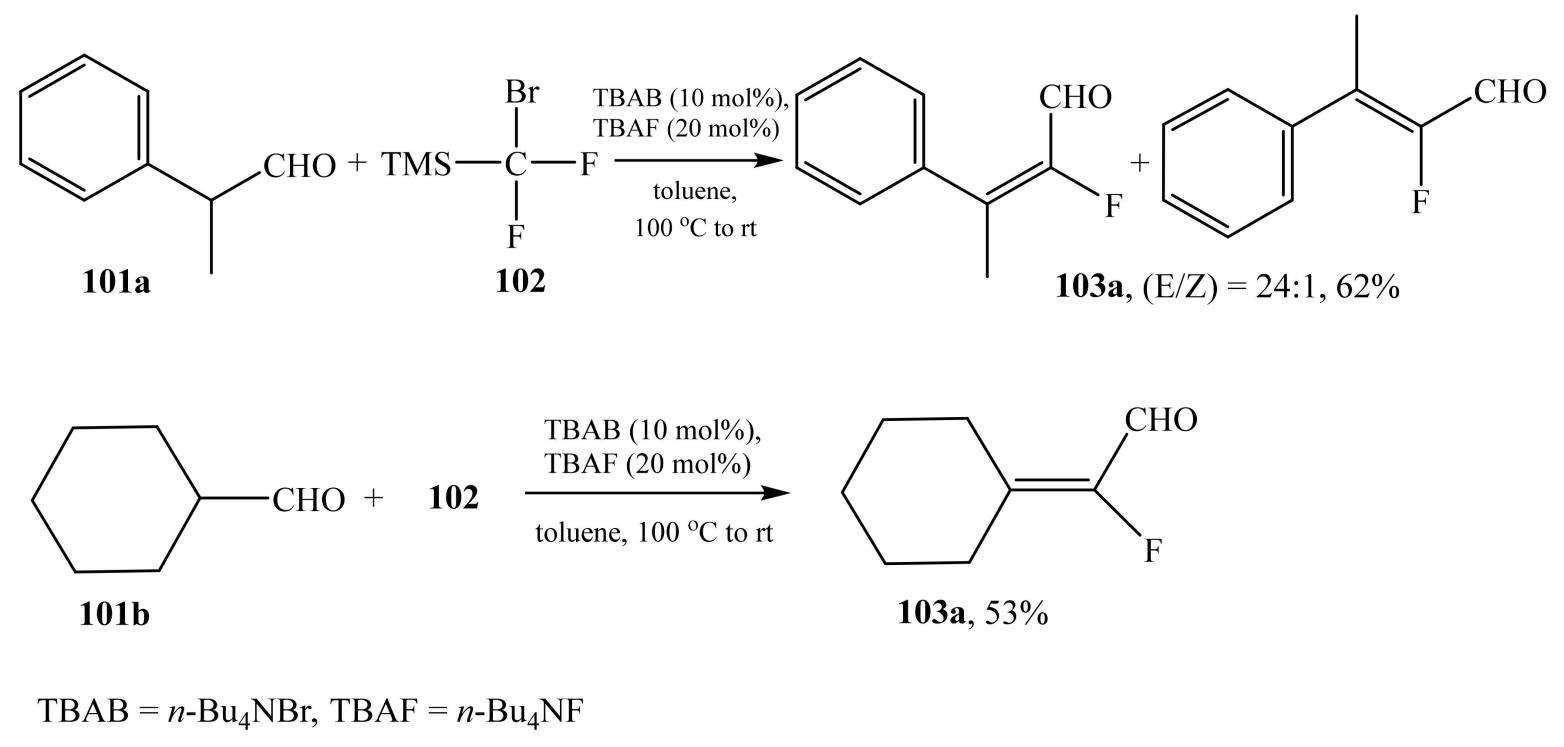
Catalytic domino reactions of aldehydes **101a,b** with TMSCF_2_Br.

Recently, Zhu et al. [101] developed a unique domino reaction of enolizable aldehydes **101a,b** with Me_3_SiCF_2_Br (TMSCF_2_Br) **102** to construct α-fluoroenals **103a,b**. (Scheme 41).

**Scheme 42:**
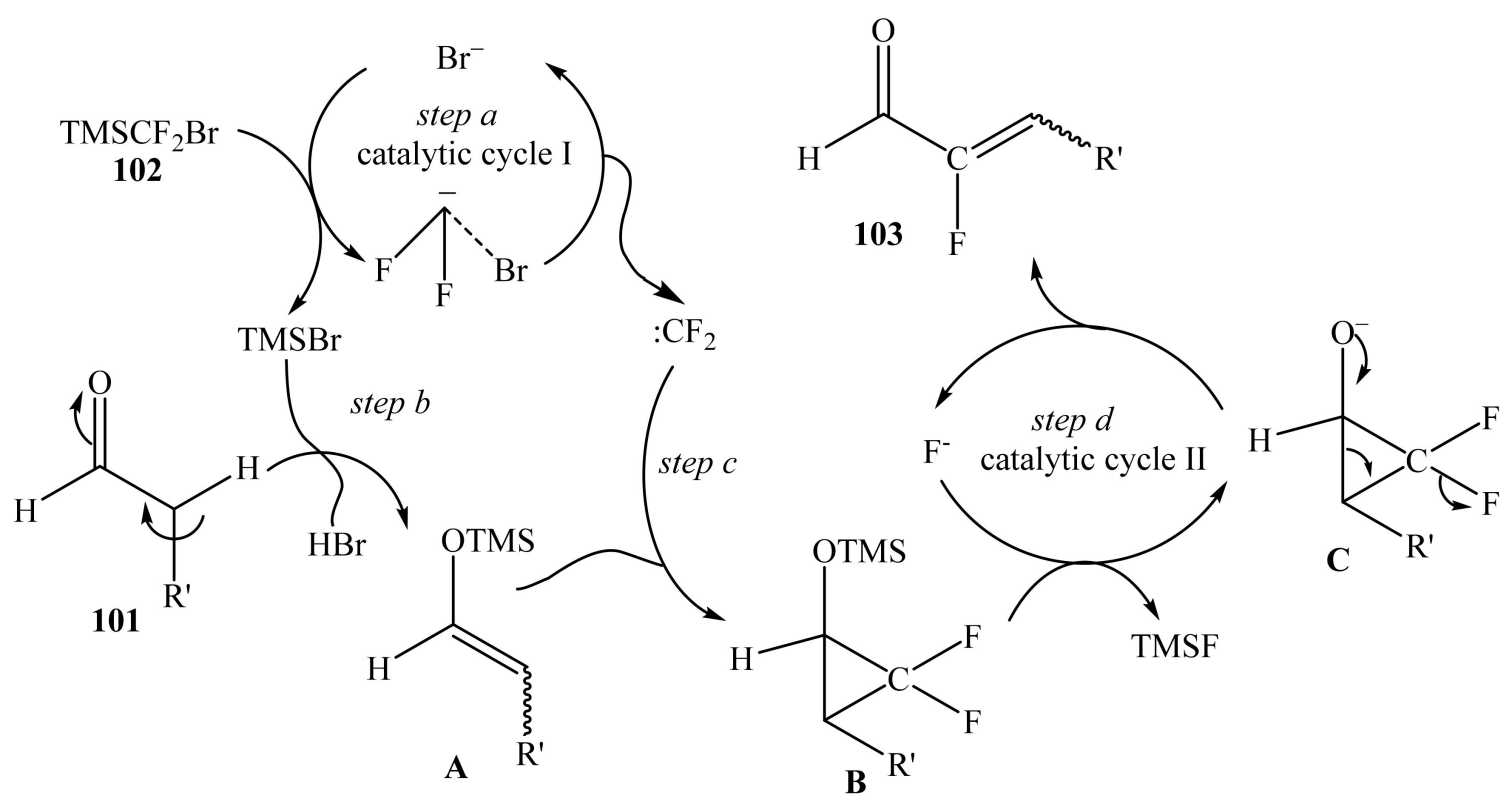
Mechanism of domino reaction of aldehydes with TMSCF_2_Br.

A tentative reaction mechanism is depicted in Scheme 42. The reaction involves in situ formation of difluorocarbene (step a) and silyl enolether **A** (step b), difluorocyclopropanation (step c), desilylation, ring-opening, and defluorination (step d). In this tandem reaction, Me_3_SiCF_2_Br acts not only as a difluorocarbene source, but also as a TMS transfer agent, as well as internal bromide and fluoride anion catalyst. It enables a smooth transformation in the presence of only catalytic amounts of *n*-Bu_4_NBr as an initiator. The cascade reaction proceeds mildly to give **103** and catalyzed by *n*-Bu_4_NBr (TBAB) to initially activate TMSCF_2_Br (Scheme 42, catalytic cycle I). In many cases, it is assisted by an external fluoride anion to accelerate desilylation of the target cyclic trimethylsilyl ether intermediate **B** (Scheme 42, catalytic cycle II).

**Scheme 43:**
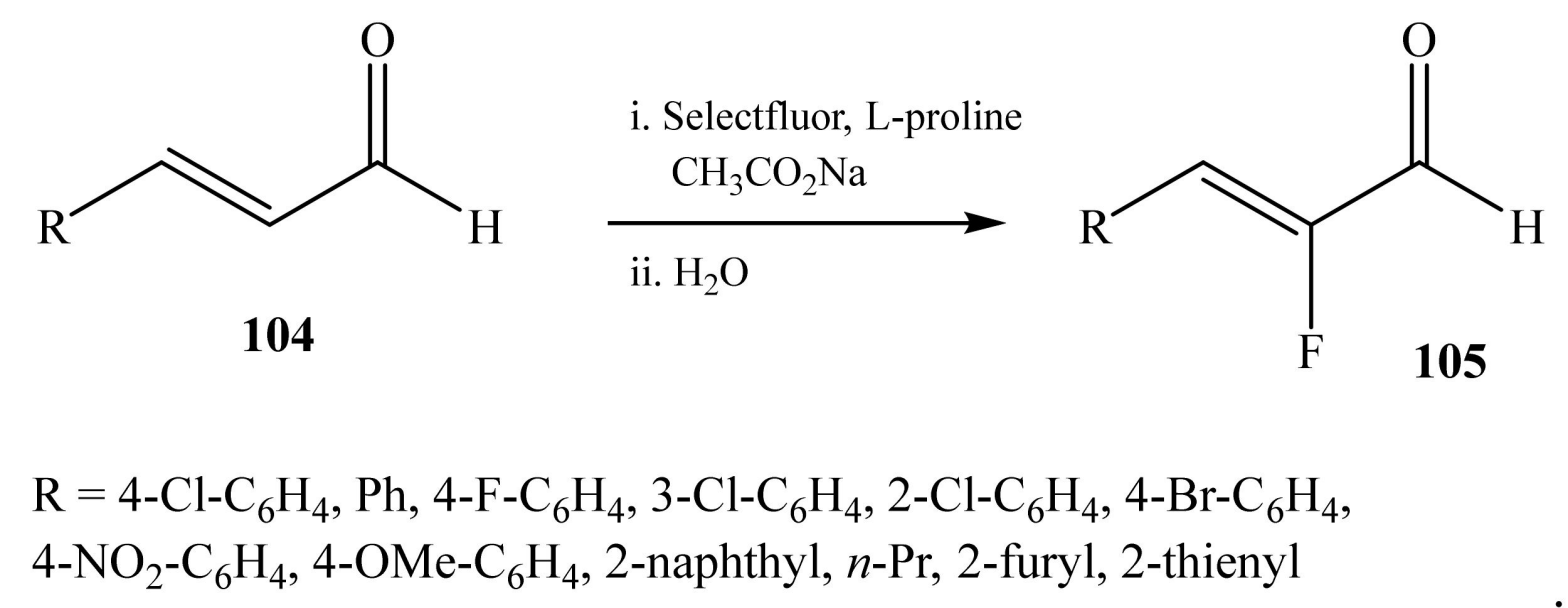
Synthesis of α-fluoro aldehydes **105**.

A one-pot, two-step L-proline-mediated stereoselective α-C(sp^2^)-H fluorination of α,β-unsaturated aldehydes **104** to deliver the corresponding (*Z*)-α-fluoro-α,β-unsaturated aldehydes **105** (Scheme 43) was documented in 2017 [102].

At the first step, Selectfluor was employed as a fluorinating agent in the CH_3_NO_2_/MeOH system to form the (*Z*)-α-fluoro-α,β-unsaturated aldehydes and their corresponding dimethyl acetals through methoxyfluorination-elimination. At the second step, water is added to promote the hydrolytic cleavage of the dimethyl acetals.

**Scheme 44:**
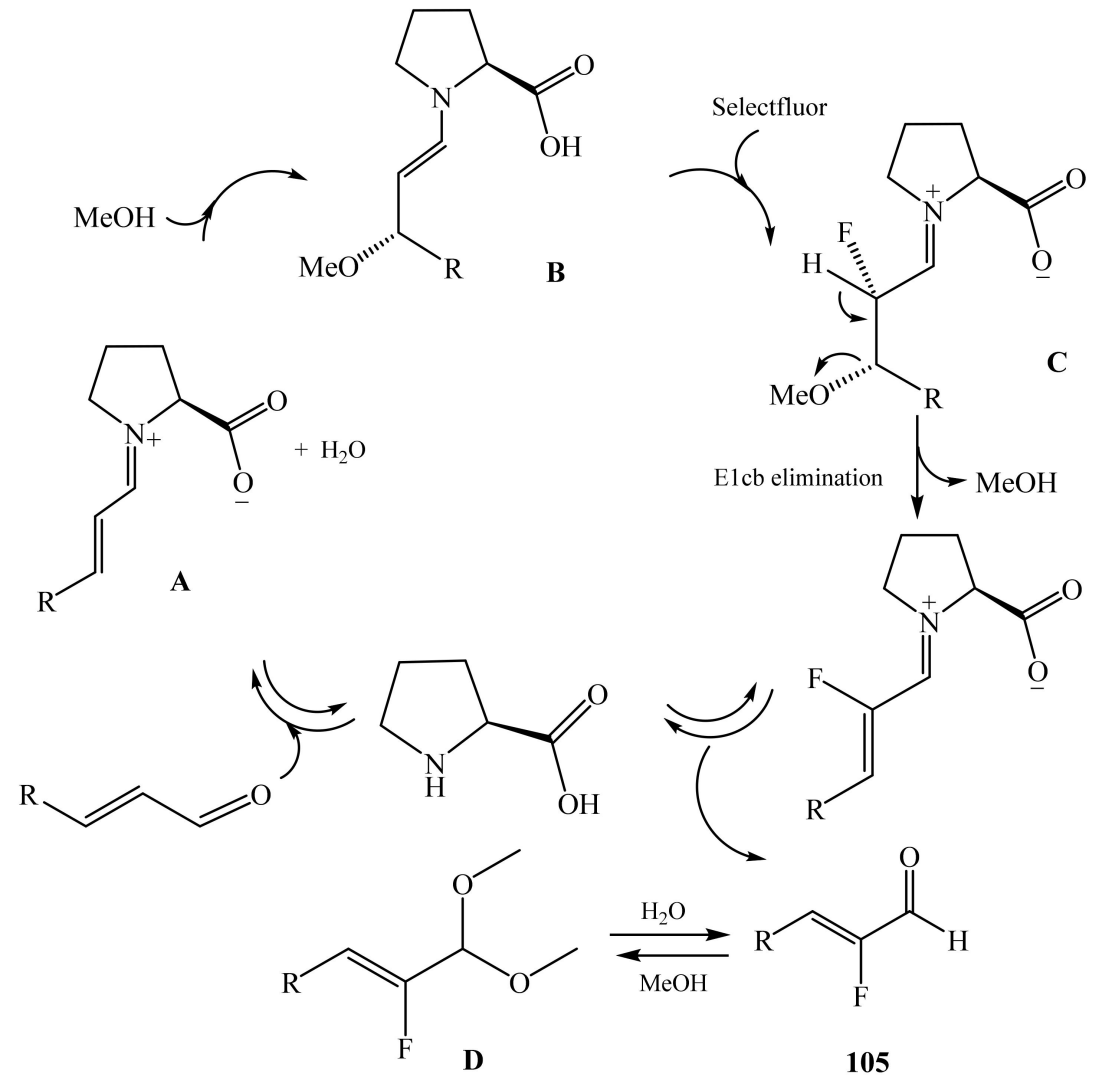
Possible mechanism of the cascade reaction of asymmetric methoxyfluorination-elimination.

Possible mechanism of the cascade reaction of asymmetric methoxyfluorination-elimination is shown in Scheme 44. The action of L-proline on aldehyde **104** generates the activated iminium **A**. The latter undergoes oxa-Michael addition of MeOH to give chiral enamine **B**. The reaction of enamine **B** with Selectfluor produces the α-fluoro-β-methoxy iminium species **C**. At last, the elimination of MeOH affords the (*Z*)-α-fluoro-α,β-unsaturated aldehyde **105** and the corresponding dimethyl acetal **D**. After complete fluorination, the addition of water promotes the hydrolytic cleavage of dimethyl acetal **D**.

The obtained (*Z*)-α-fluoro-α,β-unsaturated aldehydes **105** can be smoothly reduced to the corresponding alcohols by NaBH_4_. This method represents an expedient, facile, and mild synthetic approach to 2-fluoropropenal and 2-fluoropropenol, which are important structural units in biologically active molecules.

## 3. Conclusions

Summarizing the available literature data on the synthesis of α-functionally substituted α,β-unsaturated aldehydes, it can be concluded the classical approach to the preparation of such aldehydes via aldol condensation is still widely employed. The main shortcoming of this approach is the high probability of side processes, especially in the case of different enolizable carbonyl compounds. In this regard, considerable efforts are focused on the development of chemo- and stereoselective methods for the preparation of α-substituted alkenals.

High selectivity can be achieved using the Mannich condensation with the participation of aliphatic aldehydes and non-enolizable aldehydes in the presence of secondary amines, which act as organic catalysts generating a Mannich base in situ. Such reactions have a number of benefits, including mild conditions, high selectivity and yields, and one-pot procedure.

It should be underlined that, as α- functionally substituted α,β-unsaturated aldehydes are heterodienes, they can be obtained with high chemoselectivity via the retro-Diels–Alder reaction. In addition, other efficient and promising methods comprise various cascade transformations that combine functionalization and elimination involving aldehydes (including those already unsaturated) or their synthetic equivalents. To some extent, the lack of generality reduces the significance of these methods. Nevertheless, they continue to be developed. The application of new catalysts, two-phase systems, ionic liquids, or solvent-free protocols arouses ever-increasing interest. In addition, microwave assistance is an expedient and convenient tool to increase the yields and shorten the reaction time.

Additionally, it may be inferred that the synthetic chemistry of α-functionally substituted alkenals is rather rich, though it remains poorly studied. It is important that among functionalized unsaturated aldehydes there have been found representatives that are structurally similar to the naturally occurring compounds. This fact allows the regularities of chemical transformations that are inherent in natural substrates to be established.

Therefore, it can be expected that the generalization of information on the methods for the synthesis of α-substituted α,β-unsaturated aldehydes will contribute to the further development of chemistry of these valuable reagents, biologically active substances, ligands, and promising materials.

## Figures and Tables

**Scheme 2:**
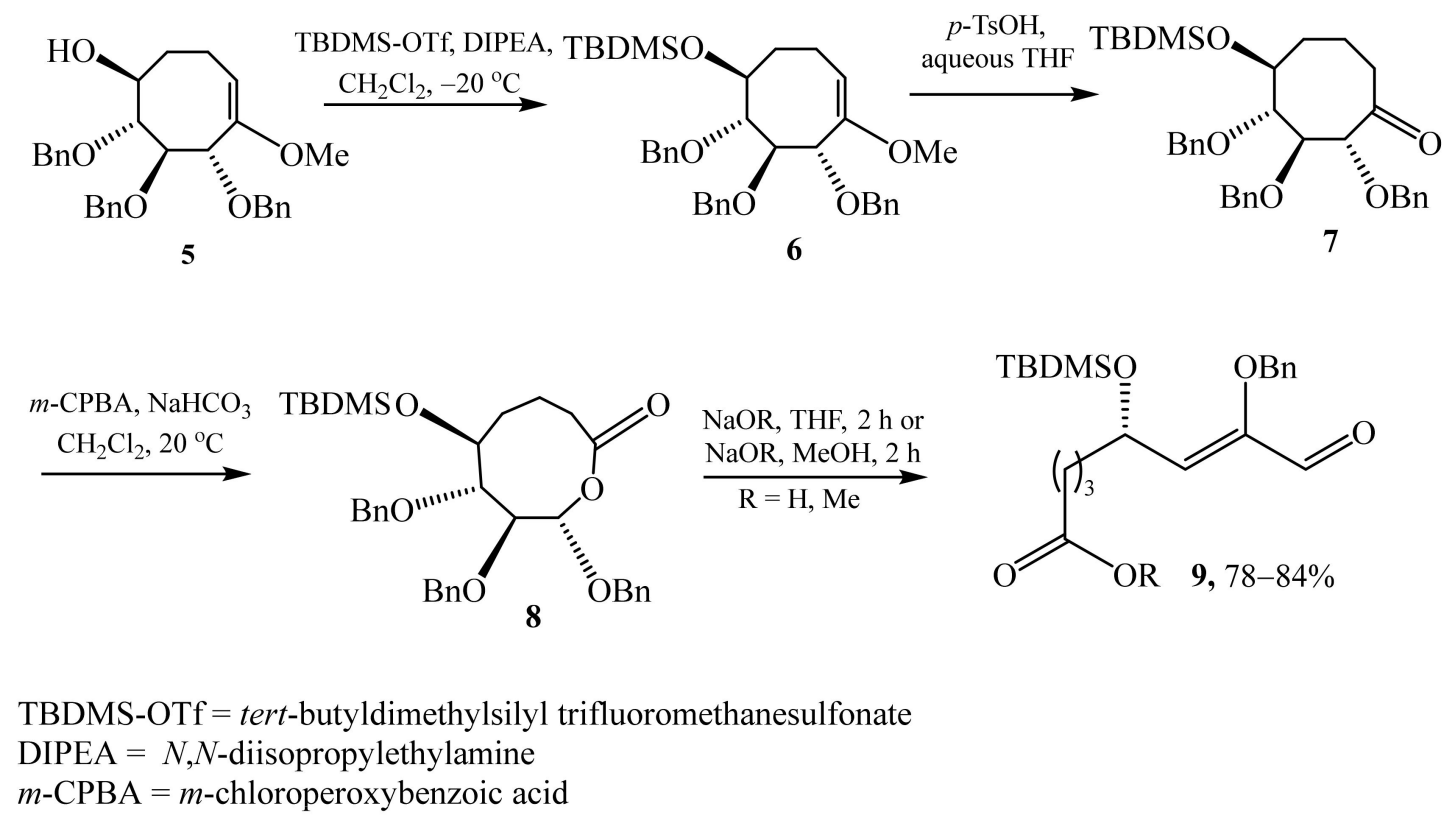
Synthesis of 2-benzyloxysubstituted 2-alkenals **9**.

## Data Availability

Not applicable.
